# The Evolutionary Potential of Phenotypic Mutations

**DOI:** 10.1371/journal.pgen.1005445

**Published:** 2015-08-05

**Authors:** Hayato Yanagida, Ariel Gispan, Noam Kadouri, Shelly Rozen, Michal Sharon, Naama Barkai, Dan S. Tawfik

**Affiliations:** 1 Department of Biological Chemistry, Weizmann Institute of Science, Rehovot, Israel; 2 Department of Molecular Genetics, Weizmann Institute of Science, Rehovot, Israel; Duke University, UNITED STATES

## Abstract

Errors in protein synthesis, so-called phenotypic mutations, are orders-of-magnitude more frequent than genetic mutations. Here, we provide direct evidence that alternative protein forms and phenotypic variability derived from translational errors paved the path to genetic, evolutionary adaptations via gene duplication. We explored the evolutionary origins of *Saccharomyces cerevisiae IDP3* - an NADP-dependent isocitrate dehydrogenase mediating fatty acids *ß*-oxidation in the peroxisome. Following the yeast whole genome duplication, *IDP3* diverged from a cytosolic ancestral gene by acquisition of a C-terminal peroxisomal targeting signal. We discovered that the pre-duplicated cytosolic *IDP*s are partially localized to the peroxisome owing to +1 translational frameshifts that bypass the stop codon and unveil cryptic peroxisomal targeting signals within the 3’-UTR. Exploring putative cryptic signals in all 3’-UTRs of yeast genomes, we found that other enzymes related to NADPH production such as pyruvate carboxylase 1 (*PYC1*) might be prone to peroxisomal localization via cryptic signals. Using laboratory evolution we found that these translational frameshifts are rapidly imprinted via genetic single base deletions occurring within the very same gene location. Further, as exemplified here, the sequences that promote translational frameshifts are also more prone to genetic deletions. Thus, genotypes conferring higher phenotypic variability not only meet immediate challenges by unveiling cryptic 3’-UTR sequences, but also boost the potential for future genetic adaptations.

## Author Summary

The rarity of genetic mutations limits the likelihood of adaptation. However, transcriptional and translational errors, so-called phenotypic mutations, are >10^5^-fold more frequent, thus generating protein mutants from unmodified genes. We provide the first evidence that phenotypic mutations paved the path to what later, after gene duplication, became newly compartmentalized enzymes. Thus, gene duplication followed rather than initiated the divergence of this new trait. Our findings also show that translational infidelity and phenotypic variability comprise the origins of evolutionary innovations, and how selection for enhanced phenotypic variability also promotes the appearance of genetic mutations that lead to the very same outcome.

## Introduction

Latent, promiscuous protein functions serve as starting points for evolving new functions, thus resolving the evolutionary ‘catch’ of no new trait can evolve unless it already exists and can confer an immediate survival benefit [1,2,3]. Along the same veins, it has been proposed that other forms of molecular infidelity, such as transcriptional and translational errors, may also underlie the evolution of new protein traits [4,5,6]. Indeed, these so-called ‘phenotypic mutations’ yield protein variability from an unmutated gene and are up to 10^5^ times more frequent than genetic mutations [7,8,9]. Phenotypic mutations may thus bridge the crucial and relatively long time gap between the appearance of a new challenge and the emergence and fixation of changes in genotype, i.e., evolutionary adaptations as often manifested in new, paraloguous genes. To this date, however, no direct evidence exists for a phenotypic mutation paving the path to a genetic, evolutionary adaptation.

Gene duplication is the source of new paralogs, including proteins with new activities or new subcellular localizations. However, different mechanisms may underlie the emergence of new functions via gene duplication. The first proposed mechanism, now known as Ohno’s model, or neo-functionalization, is initiated by duplication as a random event generating a redundant gene copy that acquires mutations under no selection. If and when a new function becomes beneficial, and if the drifted copy happens to provide this new function, the duplicated gene becomes under selection thus giving rise to a new paralog [10]. The discovery of multi-functional proteins prompted alterative models by which gene duplication follows rather than precedes the emergence of new functions. By these models, ‘gene sharing’ [11], sub-functionalization [12], or more explicitly, ‘divergence before duplication’ [13,14], the new function initially develops in the original, pre-duplicated gene. Mutations, that are largely neutral with respect to the primary, original function, may give rise to latent, promiscuous functions, which, in turn, may become under selection if and when needed [15,16]. The new function therefore becomes under selection alongside the original one, giving rise to a bi- or multi-functional protein (gene sharing). Duplication may occur at a much later stage, thus allowing the two functions to be split between two paraloguous genes (sub-functionalization).

The current literature primarily addresses how new binding, regulatory or enzymatic functions evolve via duplication, thus providing ample evidence for divergence via multifunctional ancestors [17,18,19,20,21]. However, protein function relates not only to what a protein does but also to where it functions and with which partners. In eukaryotic cells, for example, proteins localize to different subcellular compartments to perform their designated functions. Indeed, about a third of duplicate protein pairs derived from the yeast whole-genome duplication (WGD) that occurred along the lineage leading to *S*. *cerevisiae* localize to different subcellular compartments. However, the evolutionary mechanisms underlying the divergence of gene paralogues with new subcellular localizations remain largely unknown [22]. Divergence before duplication, and a subsequent sub-functionalization to two paralogues, demands the appearance of the new trait within the ancestral, pre-duplicated gene while maintaining its original function [14,23,24,25]. In the case of localization, this means dual localization, a phenomenon that is in fact well recorded [26,27,28]. Amongst other mechanisms, the partial expression of protein forms carrying targeting signal sequences may occur via alternative splicing or transcriptional/translational errors [29,30,31]. To this date, however, no particular example exists whereby a phenotypic mutation led to the divergence of a new paralog in a recently diverged species.

To study the history of evolution of new protein localizations, we sought to examine duplicate gene pairs that derived from the yeast WGD and diverged in their cellular localizations. Of the potential candidates listed in the literature [22,32,33], one gene stood out in having a clear-cut selectable phenotype, and hence being amenable to laboratory evolution experiments. *Saccaromyces cerevisiae IDP3* is an NADP-dependent isocitrate dehydrogenase that following the WGD diverged towards peroxisomal localization. Peroxisomes are ubiquitous eukaryotic subcellular compartments where oxidative reactions occur, most notably the degradation of fatty acids via *β*-oxidation. *IDP3* is selectively essential for yeast growth on unsaturated fatty acids as main carbon source, providing the reducing agent NADPH in peroxisome for the *β*-oxidation of these fatty acids such as petroselinate [34]. *S*. *cerevisiae* has three differently compartmentalized *IDP* paralogues: mitochondrial *IDP1*, cytosolic *IDP2* and peroxisomal *IDP3*. While the divergence of *IDP1* is an ancient event, *IDP2* and *IDP3* derived from the WGD and share >77% sequence identity. Indeed, in species that diverged prior to the WGD, apart from *IDP1*, a single *IDP* copy exists corresponding to cytosolic *IDP2*. We thus examined the evolutionary mechanisms that underlie the divergence of the ancestral cytosolic *IDP2* to give the newly localized peroxisomal paralogue *IDP3*.

## Results

### *IDP3* relocalized to the peroxisome by the acquisition of PTS1 motif

Peroxisomal proteins are transported from the cytosol, most commonly, as with *IDP3*, via a carboxy-terminal peroxisomal targeting signal. This signal, dubbed PTS1 primarily comprises a tripeptide motif: (S/A/C)-(K/R/H)-(L/M)-_*_; whereby * represents a stop codon [35]. While additional C-terminal residues affect targeting efficiency, the last 3 C-terminal residues are most crucial for peroxisomal targeting [36]. We first analyzed the *IDP* gene sequences from *S*. *cerevisiae*-related species that diverged before and after the WGD. The pre-WGD species posses only a cytosolic *IDP2* gene with no PTS1 signature, whereas the post-WGD species all have a peroxisomal *IDP3* paralogue with a C-terminal PTS1 as well as a cytosolic *IDP2* gene (Table 1). By this generally-accepted analysis [22], the ancestral *IDP2* was presumably localized to the cytosol, and following the WGD, *IDP3* neo-localized to the peroxisome by acquiring a PTS1 while *IDP2* remained a cytosolic isozyme.

**Table 1:** C-terminal peroxisomal target sequences (PTS1) of yeast *IDP* genes.

Species	Gene name	C-terminus^a^	PTS1 score^b^	Evaluation^c^
*S*. *cerevisiae*	YNL009W (*IDP3*)	SSNEDKKGMCKL*	5.3	Targeted
*S*. *paradoxus*	spar257-g2.1 (*IDP3*)	SSNGGKKDMCKL*	6.7	Targeted
*S*. *mikatae*	smik639-g9.1 (*IDP3*)	VVNERKKSLCRL*	4.1	Targeted
*S*. *bayanus*	sbayc655-g8.1 (*IDP3*)	MIRSSTGSMCKL*	1.4	Targeted
*C*. *glabrata*	CAGL0H03663g (*IDP3*)	EFNSHFNKPSKL*	5.0	Targeted
*S*. *castellii*	Scas695.13 (*IDP3*)	GFEKISPTRCKL*	1.4	Targeted
*S*. *cerevisiae*	YLR174W (*IDP2*)	SRLKKEFEAAAL*	-36.0	Not targeted
*S*. *paradoxus*	spar136-g12.1 (*IDP2*)	SRLKKEFEAAAL*	-36.0	Not targeted
*C*. *glabrata*	CAGL0B04917g (*IDP2*)	ENRIKSEFQKNF*	-33.2	Not targeted
*S*. *castellii*	Scas472.6 (*IDP2*)	EKRLTREFKQIF*	-31.9	Not targeted
^d^ *K*. *lactis*	KLLA0F12342g (*IDP2*)	KRLDSEFKSSFN*	-45.9	Not targeted
^d^ *A*. *gossypii*	AAL022W (*IDP2*)	RLADGYKRLFCE*	-49.6	Not targeted
		RLFVNKKKQAKL*	5.2	Targeted
^d^ *S*. *kluyveri*	SAKL0D08426g (*IDP2*)	EKRLIAAFRDEF*	-54.9	Not targeted
		FVTNFKSNLSKL*	5.5	Targeted
^d^ *K*. *thermotolerans*	KLTH0H12012g (*IDP2*)	EQRLIRSLKEDR*	-53.9	Not targeted
		VLYVALKRTARM*	1.9	Targeted
^d^ *K*. *waltii*	Kwal0.191 (*IDP2*)	ERRLHQGFKSQS*	-33.5	Not targeted
		AFIKALNPKAKL*	-0.8	Twilight zone
*C*. *lusitaniae*	CLUG_01682 (*IDP2*)	AVQERLNKNLGR*	-51.9	Not targeted
*D*. *hansenii*	DEHA0E24134g (*IDP2*)	AVAKRLNKNLGA*	-51.5	Not targeted
*C*. *guilliermondii*	CLUG_01682 (*IDP2*)	AVKVRLDKNLAK*	-47.9	Not targeted
*C*. *tropicalis*	CTRG_00909 (*IDP2*)	AVANRLNKNLGY*	-36.5	Not targeted
*C*. *albicans*	CAWG_01578 (*IDP2*)	VANRLNKNLGYA*	-44.9	Not targeted
*L*. *elongisporus*	LELG_00093 (*IDP2*)	VASRLNKNLGRS*	-27.6	Not targeted

^a^Marked in bold are the last 3 amino acids and stop codon (*) of the identified PTS1 sequences. Additional C-terminal residues may modulate targeting efficiency, with the last 12 C-terminal residues being most important.

^b^The PTS1 scores for the C-terminal sequences (last 12 residues) were obtained using the PTS1 predictor program [45]. Positive values indicate high probability of peroxisomal targeting.

^c^Targeting was evaluated by the program’s value thresholds.

^d^The scores of cryptic PTS1 on +1-shifted frame were calculated for the *IDP2* genes of pre-WGD Saccaromycetaceae species, except for *K*. *lactis* where no cryptic PTS1 found. Cryptic PTS1 were also not found in *IDP2* genes of pre-WGD species belonging to the CTG group and of post-WGD species (neither in- or out of frame).

To examine how *IDP3*’s new, peroxisomal localization diverged from a cytosolic *IDP2*, we replaced the coding and regulatory regions of *S*. *cerevisiae IDP3* with those of *IDP2*, and measured the effects on yeast growth in a petroselinate containing medium. A *ΔIdp3* strain was constructed from a wild-type strain that spontaneously adapted to growth on petroselinate. The wild-type *IDP2* and modified *IDP2* with addition of *IDP3*’s PTS1 at its C-terminus (*IDP2* ^*+CKL*^) were cloned into a chromosomal plasmid and transformed into the *ΔIdp3* strain. Whilst wild-type *IDP2* failed to complement the *ΔIdp3* growth on petroselinate, *IDP2* ^*+CKL*^ gave an *IDP3*-like growth phenotype (S1A Fig), as previously studied [37]. Like-wise, relative to the PTS1, the divergence of upstream regulatory elements is minor, as indicated by the same petroselinate growth when *IDP3*’s promoter region was replaced with *IDP2*’s (S1B Fig). These results suggest that acquisition of the PTS1 motif may have been necessary, and possibly even sufficient, to support divergence of *IDP3* from *IDP2*. It also appears that other changes in *IDP3*’s open reading frame, and changes in its regulatory regions, were less critical.

### Pre-duplication *IDP2* genes have a cryptic PTS1 motif within the 3’-UTR

**Fig 1:**
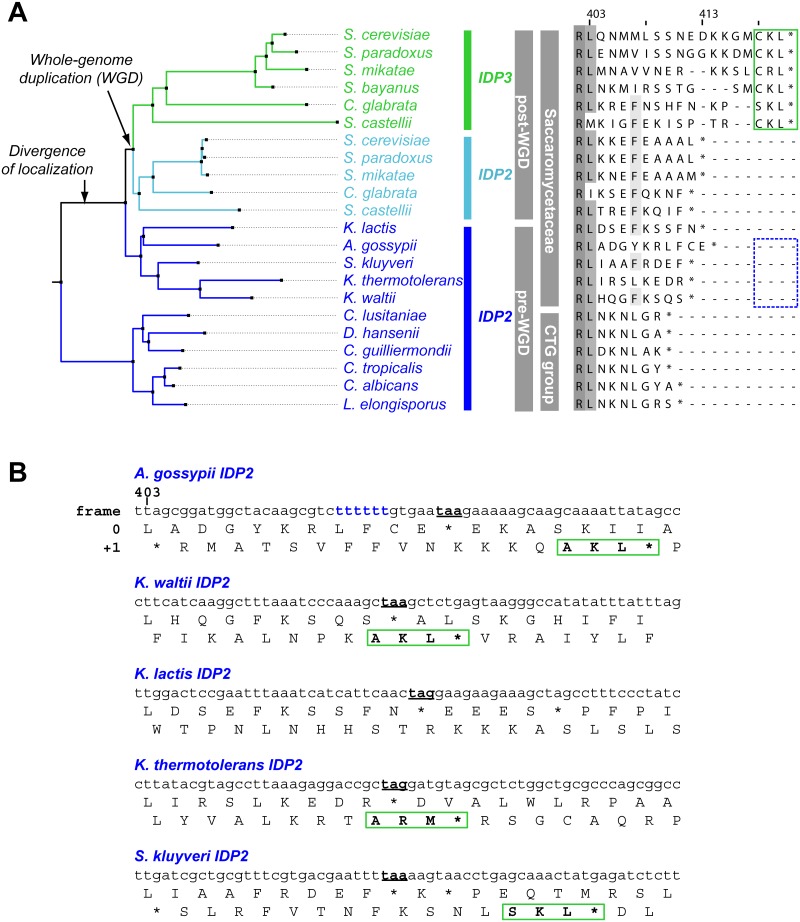
*IDP* localization diverged before the WGD via the emergence of cryptic PTS1 in the 3’UTR of the ancestral, Saccaromycetaceae *IDP2* genes. (A) A phylogenic tree and alignments of the C-termini of *IDP2* and *IDP3* genes from yeast species that diverged before and after the WGD (the alignment starts from the last conserved residue, Leu403 by *S*. *cerevisiae* numbering). Shown are pre-WGD *IDP2*s (cytosolic, in blue) and post-WGD *IDP2*s (cytosolic; light blue) and *IDP3*s (peroxisomal; green). The green rectangle marks explicit, in-frame C-terminal PTS1 signals while the blue, dotted rectangle marks genes with the cryptic 3’-UTR PTS1 signals. (B) The C-terminal sequences of the pre-WGD *IDP2* genes, including the +1 alternative reading frames. Stop codons in the original frame are marked in bold with underline. Green rectangles indicate the identified cryptic PTS1 sequences in the +1 frame. The frameshift-inducing poly T region in *A*. *gossypii IDP2* gene is highlighted.

**Fig 2:**
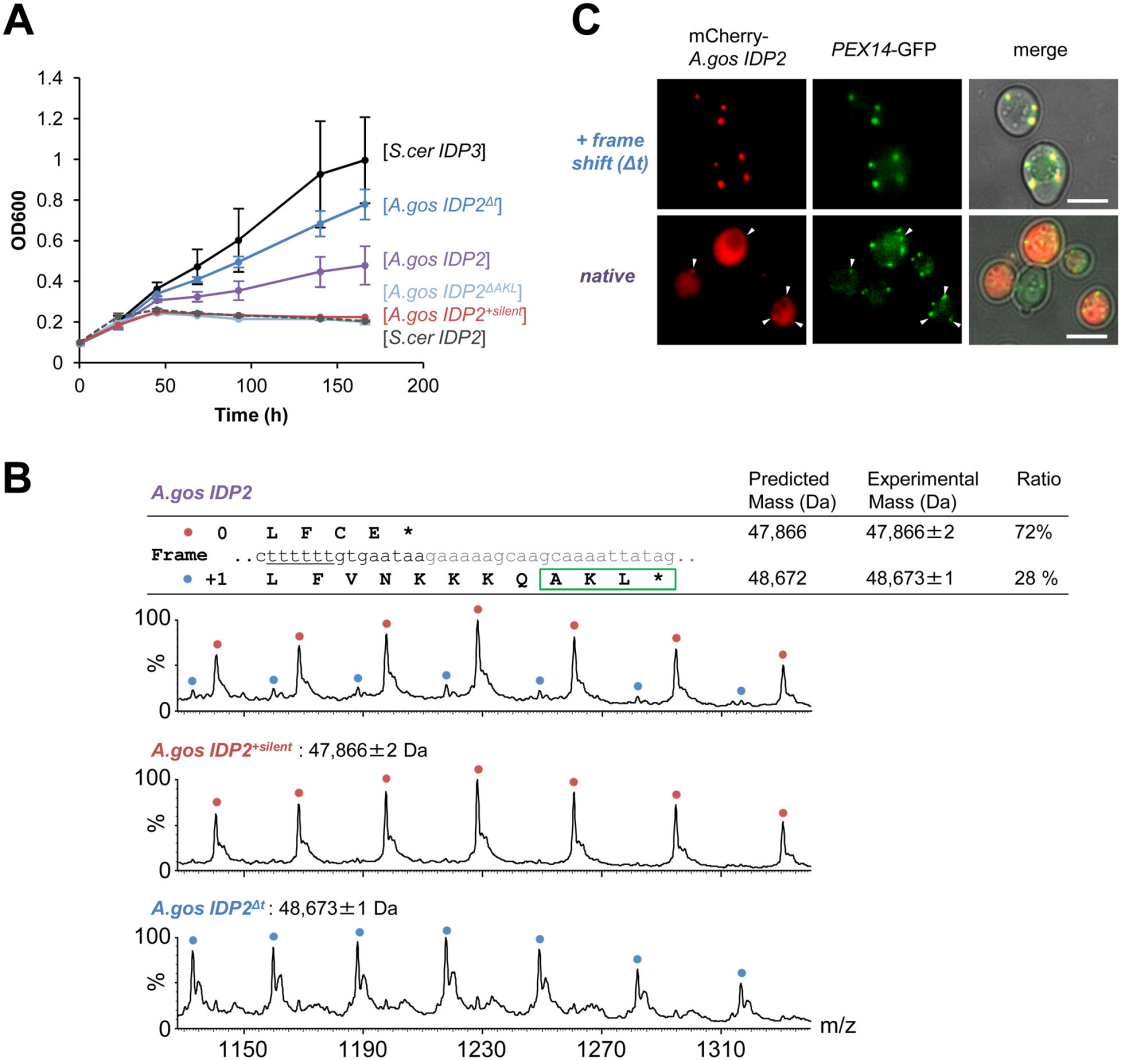
*A*. *gossypii IDP2* gene partially expresses a peroxisomal isoform by phenotypic frameshift mutation. (A) Growth of the *ΔIdp3* strain on petroselinate was complemented with the indicated genes in plasmids. *S*.*cer IDP2* shows no growth whereas *S*.*cer IDP3* promotes full growth. Wild-type *A*.*gos IDP2* partially complements, and one T deletion within its 6T repeat increases growth (*A*.*gos IDP2* ^*Δt*^). Inserting a stop codon in the cryptic PTS1 (*A*.*gos IDP2* ^*ΔAKL*^), or silent mutations in the 6T repeat (into TCTTCT; *A*.*gos IDP2* ^*+silent*^), abolished growth. (B) Wild-type *A*.*gos IDP2* and its two control mutants (*A*.*gos IDP2* ^*+silent*^ and *A*.*gos IDP2* ^*Δt*^) to express each isoform, were purified and analyzed by ESI-MS in line with liquid chromatography. The initiator Met is removed in the predicted mass. The relative intensities of original and PTS1-carrying isoforms are 72 and 28 ± 3% (n = 3). (C) Fluorescent visualization of cells co-expressing an mCherry tagged with the C-terminal *A*.*gos IDP2* fragment (Fig 1B; the last 11 amino acids and the 3’-UTR ending with AKL*) and a known peroxisomal protein, Pex14, fused to GFP. White arrows show punctate co-staining with the peroxisomal marker. The frame-shifted fragment (*A*.*gos IDP2* ^*Δt*^) having PTS1 within its coding frame was similarly tested. Scale bar, 4 μm.

We thus focused on unraveling the evolutionary origin of PTS1 motif, that is, when and how *IDP3*’s peroxisomal signal peptide emerged. By the classical Ohno’s model, the key steps towards divergence occur after duplication, and initially as drift, namely not under adaptive selection [10]. Nonetheless, we searched the C-termini and 3’-UTR sequences immediately after the stop codon of the pre-duplication *IDP2*s, attempting to identify possible starting sequences from which a PTS1 motif may have evolved via few mutations. We discovered intact, putative PTS1 motifs including an adequate stop codon located shortly after the original stop codon, in the 3’-UTRs. Putative PTS1s were found in 4 out of 5 of Saccaromycetaceae species that are phylogenetically closest to *S*. *cerevisiae* but not in more distant species including *Candida* (so-called CTG fungi group; the bottom clade in Fig 1A). However, unlike previously discovered cryptic PTS1 motifs [30,31,38], these cryptic PTS1 motifs relate not to the enzyme’s coding frame but to a +1 frameshift (Fig 1B). Accordingly, when the cryptic PTS1 was revealed in the coding frame by a single nucleotide deletion upstream to the stop codon, *A*. *gossypii IDP2* (*A*.*gos IDP2*) enabled growth of the *ΔIdp3* strain on petroselinate (Fig 2A).

Regulated translational frameshifts are known, but they typically occur at long homorepeats such as 8A that are not observed in the coding sequences prior to the stop codons of the pre-duplication *IDP2*s. Do, then, the cryptic PTS1 motifs within the 3’-UTR regions comprise relics of ancestral PTS1 motifs that were non-functionalized; or do they still encode a functional peroxisomal targeting signal and are thereby maintained under selection for dual localization? The latter seems likely given that sequences that perfectly match a functional PTS1 (Table 1 and Fig 1B) are found in 4 out of 5 of the pre-WGD Saccaromycetaceae species. To investigate the possibility that the pre-duplication *IDP2* genes partially produce a peroxisomal isoform carrying a PTS1 via a transcriptional or translation frameshift, we tested whether wild-type *A*.*gos IDP2* can enable petroselinate growth of the *S*. *cerevisiae ΔIdp3* strain. Indeed, growth on petroselinate could be observed with wild-type *A*.*gos IDP2* at about half the rate observed with *S*. *cerevisiae*’s original, peroxisomal *IDP3*, and mutating the cryptic PTS1 within the 3’-UTR abolished the growth (Fig 2A). Thus, a peroxisomal *IDP* isoform carrying the PTS1 motif seems to be co-expressed alongside the original, cytosolic form.

How are the cryptic PTS1 recruited in the coding region? Homonucleotide repeats show consistently higher tendency for slippage of RNA polymerases, and the ribosome, thus inducing phenotypic frameshift mutations [39,40,41]. Indeed, a 6T repeat exists shortly before the original stop codon of *A*.*gos IDP2* and within a highly diverged segment of the C-terminus (*S*.*cerevisiae* Leu403 is the last conserved position in *IDP* alignments; Fig 1B). Accordingly, silent mutations replacing 2 out of the 6 T within this repeat gave no complementation (Fig 2A).

We further examined whether the phenotypic frameshift at the 6T repeat is due to a transcriptional or translational error. Total RNA from *S*. *cerevisiae* expressing *A*.*gos IDP2* and grown on petroselinate was extracted. The cDNA derived from mRNAs of *A*.*gos IDP2* gene was amplified by RT-PCR and cloned for sequencing. In randomly picked 36 clones, all carried a 6U site, corresponding to the original gene’s 6T sequence, and no other sequence changes were detected along 500 bp flanking *IDP2*’s stop-codon. The phenotypic frameshift is therefore caused by translational errors, consistently with the fact that they are ~10-fold more frequent than transcriptional errors [9]. Overall, these results suggest that the *A*. *gossypii IDP2* partially produces an alternative isoform carrying a PTS1 motif via translational error that bypasses the stop codon and unveils the cryptic, frame-shifted PTS1, and thus exhibiting peroxisomal *IDP* activity that enables growth on petroselinate.

We further validated the coexistence of two isoforms of *A*.*gos IDP2* by mass spectrometry. Alongside the expected *A*.*gos IDP2* gene product, a higher mass form corresponding to the predicted frame-shifted product at the 6T repeat including the C-terminal AKL was observed with ~30% of the total mass (Fig 2B). Peroxisomal targeting by the cryptic PTS1 was also observed by fluorescent cell imaging. Red fluorescent protein (mCherry) was C-terminally tagged with the C-terminal fragment of *A*.*gos IDP2* containing the cryptic PTS1 in the 3’-UTR. We observed clear punctate co-staining with the peroxisomal marker protein Pex14 fused to GFP when the cryptic PTS1 was revealed by the frameshift (one T deletion in the 6T repeat). On the other hand, wild-type *A*.*gos IDP2* fragment fused to mCherry was primarily visualized in the cytosol, yet with also weak, punctate co-staining with the peroxisomal marker, likely indicating dual localization (Fig 2C).

We subsequently tested the cryptic PTS1 motifs of the other pre-duplication *IDP2* genes. The C-terminus of *S*.*cer IDP2* was replaced with the C-termini of 5 different pre-duplication *IDP2* genes, including their 3’UTRs (Fig 1B). As expected, *K*. *lactis IDP2* that contains no cryptic PTS1 showed no growth complementation. Three pre-duplication *IDP2*s containing the cryptic PTS1 appeared to partially express peroxisomal isoforms at a level similar to *A*.*gos IDP2*, although only *A*.*gos IDP2* has a >3 bp long homorepeat site (S2A Fig). In contrast, *K*. *waltii IDP2* (*K*.*wal IDP2*) failed to show complementation despite the existence of the cryptic PTS1 motif. Nonetheless, growth rate on petroselinate was significantly enhanced when the cryptic motif was revealed by a genetic frameshift (one nucleotide deletion before the stop codon; S2B Fig). We subsequently tested complementation with the full length of *K*.*wal IDP2* open-reading-frame including 150 bp downstream after the stop codon. The full-length *K*.*wal IDP2* showed complementation although with lower growth rates than the other pre-duplication *IDP2*s. Thus, although *K*. *waltii IDP2* appears to have a functional cryptic PTS1, unveiling it by a phenotypic frameshift seems to be dependent on having the broader context of the *K*. *waltii* gene and not just the region around the stop-codon, as was the case with the other pre-duplication *IDP2* genes. The frequency of ribosomal slippage may therefore depend on the secondary structure of mRNA, as well as on environmental factors that regulate translational fidelity in the host organism [42,43].

### How prevalent are cryptic peroxisomal signals in the yeast genomes?

**Fig 3:**
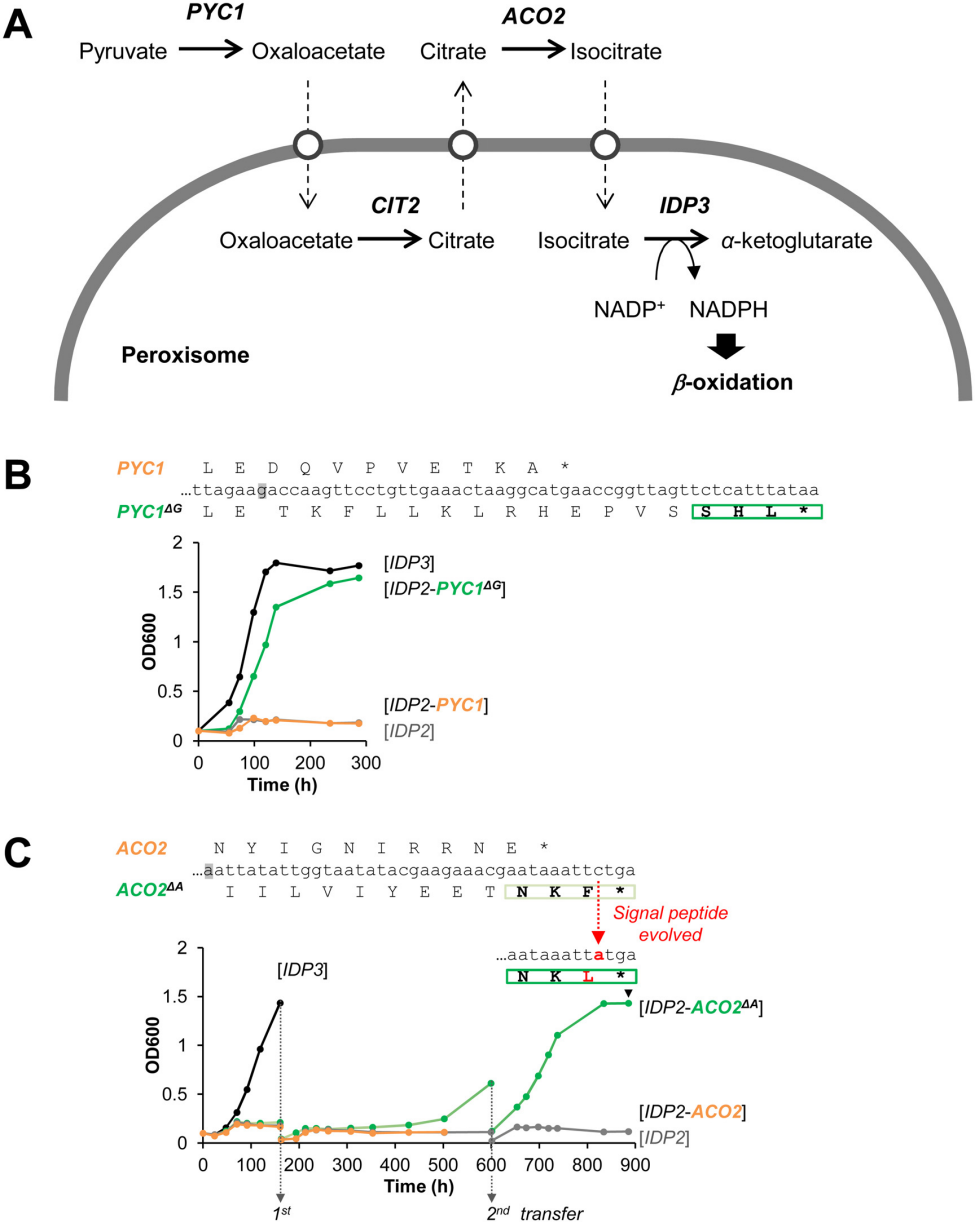
The potential peroxisomal localization of a pathway supplying NADPH for *β*-oxidation. (A) The putative pathway follows the TCA reaction cycle, beginning with pyruvate and comprising four enzymes: *PYC*, pyruvate carboxylase; *CIT*, citrate synthase; *ACO*, aconitase; and finally *IDP*, isocirate dehydrogenase. Two enzymes have peroxisomal *S*. *cerevisiae* paralogous:—*CIT3* and *IDP3*; the other two, *PYC* and *ACO*, are thought to act in the cytosol and mitochondria, respectively. Hypothetical transporters permitting the export and import of intermediate metabolites are depicted as white circles. (B) The PTS1-like motif of *S*.*cer PYC1* (last 11 amino acids *plus* the 3’UTR ending with SHL*) was tested by fusion to the C-terminus of *S*.*cer IDP2* and complementation of *ΔIdp3* growth on petroselinate. Shown is growth with the original motif (orange line), and a version obtained by a single base deletion upstream the stop codon (green line) that puts the putative PTS1 in-frame (*PYC1* ^*ΔG*^; the deleted G is highlighted in grey). (C) The PTS1-like motif of *S*.*cer ACO2* (last 10 amino acids *plus* the 3’UTR ending with NKF*) was tested as described in part (B) above. An in-frame version obtained via a deletion of a single base upstream the stop codon (*ACO2* ^*ΔA*^) was also tested. Whilst the initial growth was very slow, upon serial transfers to fresh petrosalinate media (5-fold dilutions) a marked increase in growth rate was observed. Single colonies were randomly isolated from the 2^nd^ transferred culture (at 900 h) and sequenced. All 7 sequenced clones possessed a single mutation in the PTS1-like motif converting it to NKL (S4C Fig).

Following the above findings, and a report that appeared while this work was ongoing on cryptic peroxisomal targeting of two cytosolic enzymes [30], we performed a systematic computational search of the 3’-UTR regions of four closely related post-WGD *Saccharomyces* genomes: *S*. *cerevisiae*, *S*. *paradoxus*, *S*. *bayanus* and *S*. *mikatae* (S1 and S3 Tables). The search was based on PTS1 motifs containing all possible variation of amino acids ((S/A/C/E/I/H/Q)-(K/R/H)-(L/F)-stop) from 20 peroxisomal proteins in the Saccaromyces genome database. We searched for the motif starting up to 30 bp downstream the stop codon in all frames. PTS1-like motifs were found in around 1% of total genes of the genomes. However, about 40% of these were interrupted by another stop codon (S1 Table). Further, only a small number of these potentially cryptic motifs were found in more than one species, suggesting that these motifs are under functional selection. We thus focused on few interesting candidates that are conserved among the post-WGD species, and foremost on pyruvate carboxylase 1 (*PYC1*)—a cytosolic enzyme converting pyruvate to oxaloacetate. The NADPH used for peroxisomal *β*-oxidation could be produced from pyruvate by a putative pathway that includes four enzymes: *PYC*: pyruvate carboxylase; *CIT*: citrate synthase; *ACO*: aconitase; and finally *IDP*: isocirate dehydrogenase. Although not established as a peroxisomal NADPH providing pathway, this reaction sequence comprises part of the TCA cycle. Among these, two enzymes have known peroxisomal paralogues in *S*. *cerevisiae*: *CIT2* and *IDP3*. The other two, *PYC* and *ACO*, are thought to act in the cytosol and mitochondria, respectively, thus demanding the shuttle of their substrates and products to and from the peroxisome (Fig 3A) [44]. We identified, however, a cryptic PTS1-like motif (SHL*) in the *PYC1* genes of all four *Saccharomyces* species. The motif of *S*. *cerevisiae PYC1* is located at 11 bp downstream from the stop codon, in a +1-shifted frame, and predicted as a weak motif by the PTS1 predictor [45] (S3A Fig).

We examined the functionality of the PTS1-like motif of *S*.*cer PYC1* by tagging *S*.*cer IDP2* at the C-terminus with the C-terminal fragment of *S*.*cer PYC1* (the last 11 amino acids and the 3’UTR ending with SHL*). The *PYC1* motif showed functional targeting when recruited within the coding frame via a single nucleotide deletion, as indicated by *ΔIdp3* complementation for growth on petroselinate, while not functional with the native sequence (Fig 3B). Peroxisomal localization was also observed by fluorescent imaging with mCherry C-terminally-tagged with the 3’UTR motif revealed by a single nucleotide deletion (S3B Fig). These results suggest that this motif is relevant for peroxisomal targeting of *S*.*cer PYC1* via phenotypic errors. At a minimum, our results indicate that a duplicated *S*.*cer PYC1* is within a single genetic mutation from becoming a functional peroxisomal paralog, or perhaps that *PYC1* was dually localized in the past.

Our computational search did not identify consensus PTS1 motifs ((S/A/C/E/I/H/Q)-(K/R/H)-(L/F)-stop) in *ACO* genes. However, upon a closer look we identified a PTS1-like motif (-NKF*) located at +1-shifted frame shortly after the stop codon of *S*.*cer ACO2* (S4A Fig). This motif gave very weak functional targeting when inserted in-frame at the C-terminus of *IDP2* and tested for growth on petroselinate (S4B Fig). However, in a continuous passage culture, the slow growth was dramatically accelerated and eventually matched the growth rate of *IDP3* (Fig 3C). Sequencing of randomly chosen clones from the petroselinate culture identified a single nucleotide exchange that occurred spontaneously, converting NKF* to NKL* and thus yielding a stronger targeting signal (S4C Fig). The rapid fixation of this mutation demonstrates the ease by which the latent *ACO2* motif can further evolve to yield an efficient targeting signal.

### Phenotypic and genetic mutations are correlated

How do phenotypic mutations, *e*.*g*. the slippage in pre-duplication *IDP2*s, become eventually ‘imprinted’ via a genetic mutation, thus leading to evolutionary adaptation as observed in the extant, peroxisomal *IDP3*s? Homonucleotide repeats of 3–8 bases are prone to phenotypic, transcriptional/translational errors as exemplified here with *A*. *gossyppii IDP2* (Fig 2B). However, homorepeats are also highly prone to genetic, frame-shifting InDels (insertions and deletions). In fact, these two phenomena are strongly correlated: the longer the homorepeat, the higher is the frequency of both phenotypic and genetic frameshifts (see Ref. [39] and references therein). However, apart from *A*. *gossyppii IDP2*, the 3 other pre-duplication *IDP2* genes have no homorepeats of >3 bases length in the region before the stop-codon (Fig 1B). We therefore sought to identify the sites of slippage that unveil the cryptic PTS1 sequences, and to also establish whether the very same sites also comprise hotspots for genetic deletion mutations that result in the exclusive expression of a peroxisomal form.

**Fig 4:**
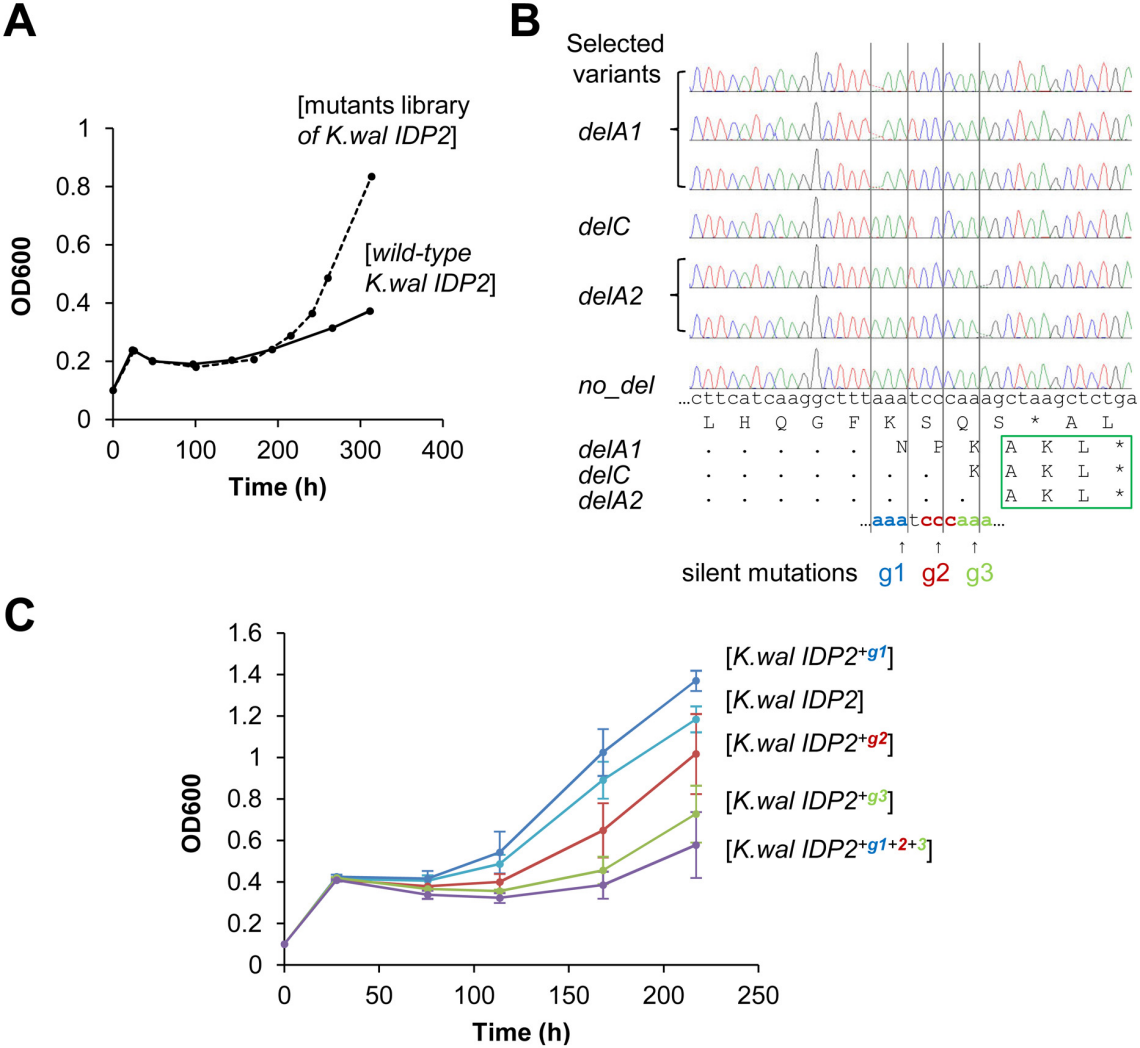
Laboratory evolution for peroxisomal localization is driven by genetic mutations at the same location where phenotypic mutations occur. (A) The C-terminal region of *K*. *waltii IDP2* including the 3’-UTR (100 bp around the stop codon) was randomly mutated by error-prone PCR at an average of ~1 mutation per gene. The mutated genes were cloned and selected by plasmid complementation of the *ΔIdp3* growth in YP medium with petroselinate. After 200 hours of culture, a burst in growth rate was observed. (B) Six out of seven randomly sequenced clones from the 300 hours culture had a single base deletion just before the stop codon (g1-g3), all resulting in an in-frame PTS1 signal (-AKL*). (C) The three deletion sites were modified by silent mutations of the wild-type base into G. The resulting mutants were tested by plasmid complementation of the *ΔIdp3* strain for growth on petroselinate. Two silent mutations (g2 and g3) resulted in growth inhibition, and the triple mutant (*K*.*wal IDP2* ^*+g1+2+3*^) showed essentially no growth, thus indicating that the translational slippage leading to dual localization of *K*. *waltii IDP2* occurs within this segment. It should be noted that we used an adapted *ΔIdp3* strain whereby the growth of *ΔIdp3* complemented with *K*.*wal IDP2* increased (see Materials and Methods).

In fact, we began our exploration with the latter—namely, we sought to identify hotspots for genetic, single base deletions that may occur upstream to *K*. *waltii IDP2’s* stop-codon and result in its cryptic PTS1 becoming in-frame (*K*. *waltii IDP2* was the most poorly bypassed pre-duplication *IDP2*; and, as mentioned above, has no >3 bases repeats in its C-terminus; Fig 1B). We randomly mutated the segment of 100 bases around *K*. *waltii IDP2*’s stop-codon, transformed the mutated gene library to the *S*. *cerevisiae ΔIdp3* strain and selected the transformed yeast cells for growth on petroselinate. After 200 hours, the culture’s growth rate dramatically increased (Fig 4A). The selected pool was analyzed by sequencing seven randomly chosen clones. We identified 3 different single base deletions that all occurred within a stretch comprising 3 repeats of 3 bases each just before the stop codon (AAATCCCAAA; Fig 4B). To examine whether the phenotypic frameshifts occur within the very same stretch, we applied the same test applied to validate the 6T repeat as the site of ribosomal slippage in *A*. *gossyppii IDP2*. Namely, we introduced silent mutations at each of the 3 deletion sites (AA**A** TC**C** CA**A** A; in bold, the sites of silent mutations; Fig 4B) and examined whether the frequency of slippage, as reflected by the rate of growth on petroselinate, would be reduced. Indeed, silent mutations in the two deletion sites that are closer to the stop-codon showed a marked inhibition of growth, and the triple mutant showed effectively no growth (Fig 4C). It therefore appears that the phenotypic mutations leading to cryptic peroxisomal localization in the cytosolic *IDP2*s are readily ‘immortalized’ via genetic deletion mutations that occur within the very same site.

## Discussion

Taken together, our results show that *S*. *cerevisiae IDP3* diverged from an ancestral, pre-duplicated gene that, although primarily localized to the cytosol, had the capacity for peroxisomal localization via a phenotypic mutation—a frameshift induced by translational slippage. We appear to be witnessing all the putative intermediates along this evolutionary trajectory. Specifically, *S*. *cerevisiae IDP3*, and the other post-duplication *IDP3*s, all have a ‘legitimate’ in-frame PTS1. The pre-duplication cytosolic *IDP2*s in Saccaromycetaceae species have a cryptic PTS1 within their 3’-UTR regions that are unveiled by translational and/or transcriptional errors. Further, the contemporary *S*. *cerevisiae PYC1* and *ACO2* genes appear to contain cryptic PTS1 signals in their 3’-UTRs that are readily revealed by a single genetic deletion mutation. Indeed, our findings also suggest that four enzymes, that together comprise a putative pathway providing NADPH for peroxisomal *β*-oxidation, either have a known peroxisomal paralogue (*CIT* and *IDP*), or have been partially localized to the peroxisome in the past or are evolving towards peroxisomal localization (*PYC* and *ACO*). Finally, our laboratory evolution experiments confirm that the pre-duplication *IDP2*s carrying cryptic PTS1 sequences readily evolve via genetic mutations to yield ‘legitimate’ peroxisome-targeted genes.

We also conclude that *IDP3*’s mechanism of divergence does not fit Ohno’s model, namely, neo-functionalization/localization. Rather, *IDP3*’s peroxisomal targeting emerged in the ancestral Saccaromycetaceae species long before the *IDP* gene was duplicated to give the newly diverged *IDP3*. As shown here, 4 out of the 5 pre-duplication *IDP2*s in Saccaromycetaceae species are dually localized via a cryptic PTS1 whilst the more distant species do not possess such cryptic PTS1s (Fig 1). Thus, *IDP*s represent a clear case of divergence via ‘gene sharing’ [11,21] and of ‘divergence before duplication’ [13,14]. Following duplication, the ancestral dual, cytosol-peroxisome localization function was split between two paralogous genes, thus representing a case of sub-functionalization/localization [22,24]. The latter evolutionary mechanisms rely on one gene executing multiple functions, and accordingly on weak trade-off—namely, that mutations that endow the newly emerging function do not abolish the original function. Weak trade-offs, *i*.*e*., ‘something for nothing’, at least at the early stages of evolution, is a key feature that makes divergence before the duplication a far more plausible scenario than Ohno’s model [46]. Indeed, the assumption of tradeoffs underlies Ohno’s model—the existence of a redundant copy relieved from the burden of selection enables mutations to freely accumulate, including mutations that undermine the original function [47]. Our analysis indicated a ratio of ~1:3 of the peroxisome-cytosol isoforms in *A*. *gossyppii IDP2* (Fig 2B). Thus, a reduction of ~25% in the levels of the cytosolic *IDP* is enough to shift from no growth on petrosalinate to a growth rate that is only half of that observed with the ‘legitimate’ peroxisomal *IDP3* (Fig 2A). However, growth levels comparable to wild-type were afforded only upon a genetic mutation that leads to exclusive peroxisomal targeting. Assuming that cytosolic *IDP* is essential, such a mutation could only follow duplication.

*IDP*s’ divergence prior to duplication was driven by transcriptional /translational errors that result in dual localization to both the cytosol and peroxisome from a single gene. Dual localization is commonly observed (see also refs [27,28,48,49]. Specifically, stop-codon read-through, or alternative splicing, were previously shown to mediate the dual cytosol-peroxisome targeting of several glycolytic enzymes in various yeast species [30]. However, there is no evidence indicating that these genes duplicated and diverged into ‘legitimate’ peroxisomal paralogues, as is the case with the pre-duplication *IDP2*s. Divergence before duplication may apply to localization signals other than PTS1. Most protein localizations, such as to the endoplasmic reticulum, mitochondria, or chloroplasts, are mediated by N-terminal target signals that are ~20 amino acids long. Translational errors can also produce various N-terminal isoforms from a single mRNA owing to alternative translation initiation sites (“leaky scanning”) [50] thus enabling dual targeting [27,29,51].

Foremost, our results provide unequivocal evidence that phenotypic mutations led to the evolution of new traits [43,52]. Noise and infidelity in general, and transcriptional and translational errors specifically, may comprise a “look-ahead” effect [6] thus underlining the phenotype of the yet to emerge duplicated, diverged gene [5]. The rate of phenotypic mutations is >10^5^-fold higher than genetic mutations, and may be further enhanced under stress due to the malfunction of translational fidelity [42], or under the yeast prionic state ([PSI+]), thus promoting phenotypic diversity that mediates survival in challenging environments [53]. Further, although phenotypic mutations are not inherited as such, the capacity to induce them is inherited via DNA sequences that favor slippage, as manifested, for example, in the 6T repeat inducing dual localization of *A*. *gossyppii IDP2*.

Finally, our results indicate another intriguing aspect of phenotypic mutations—the same gene context is prone to both phenotypic mutations (transcriptional/translational errors) and genetic mutations (Fig 4). Thus, selection for dual localization, hence favoring gene sequences whereby slippage downstream the stop-codon occurs at relatively high frequency (as seems to be the case in the pre-duplication *IDP2* genes) also creates a hotspot for a genetic mutation. In this manner, a coincidental error becomes a ‘frozen accident’ under selection, as well as a hotspot for evolutionary adaptation.

## Materials and Methods

### Yeast strains

All strains were derived from By4741 (*MATa*, *his3Δ1*, *leu2Δ0*, *met15Δ0*, *ura3Δ0*) [54]. The strains used are listed in S2 Table. To obtain the wild-type strain harboring a selection marker (*WT*; *idp3*:: *IDP3-kanMX4*), the *IDP3* open reading frame (ORF) of the By4741 genome was replaced with the *IDP3* gene fusing to kanMX4 cassette by homologous recombination [55]. This strain (*WT*) was subjected to serial passage culture in the petroselinate containing medium (1% yeast extract, 2% Bacto-peptone, 0.2% Tween-40, 0.1% petroselinate) containing G418 (200 μg/ml)) until spontaneously adapting and exhibiting higher growth rate. The *IDP3* knockout strain (*ΔIdp3*; *idp3*:: *hphNT1*) was constructed from this adapted strain, whereby the locus containing the *IDP3* gene—the kanMX4 cassette was replaced with a hpnNT1 cassette by homologous recombination.

### Cloning procedures

The kanMX4 cassette was PCR amplified from a pBS7 vector (Yeast resource center). The *IDP3* gene—kanMX4 fusion was constructed as follows: The *IDP3* gene, including the ORF and 150 bps downstream, was PCR amplified from genomic DNA. The amplified gene was introduced into the pBS7 vector containing kanMX4 cassette by using the *SmaI* /*BglII* sites. The *IDP3* gene—the kanMX4 cassette was thus PCR amplified from the sub-cloned pBS7 vector. The hpnNT1 cassette was amplified from pRS41H plasmid [56] using primers franking the 5’ end of *IDP3*’s ORF and the 3’ end of the kanMX4 cassette. DNA fragments encompassing the coding region, plus the 500 bps upstream (5’) and 150 bps downstream regions (3’) of the various *IDP* genes (*S*. *cerevisiae IDP3*, *IDP2*, *A*. *gossypii IDP2*, and *K*. *waltii IDP2*) were amplified from genomic DNA. For testing the effects of the coding vs. 5’-UTR (promoter) and 3’-UTR (cryptic PTS1s), the coding and non-coding fragments were separately amplified and combined by assembly PCR. These assembled fragments were subcloned by *SmaI/NotI* sites into pRS41K [56], a centromere-based plasmid for single-copy expression in yeast, generating: pRS41K-*ScIDP3*_*Pro*_ */ScIDP3/ScIDP3*_*Ter*_, pRS41K-*ScIDP2*_*Pro*_ */ScIDP3/ScIDP3*_*Ter*_, pRS41K-*ScIDP3*_*Pro*_ */ScIDP2* ^*+CKL*^ */ScIDP3*_*Ter*_, pRS41K-*ScIDP3*_*Pro*_ */ScIDP2/ScIDP2*_*Ter*_, pRS41K-*ScIDP3*_*Pro*_ */AgIDP2/AgIDP2*_*Ter*_, pRS41K-*ScIDP3*_*Pro*_ */KwIDP2/KwIDP2*_*Ter*_; whereby *ScIDP3*_*pro*_ and *ScIDP2*_*pro*_ are 500 bp upstream regions of *S*. *cerevisiae IDP3* and *IDP2*, respectively; *ScIDP2*, *ScIDP2* ^*+CKL*^, *ScIDP3*, *AgIDP2*, and *KwIDP2* are coding region; *ScIDP2*_*ter*_, *ScIDP3*_*ter*_, *AgIDP2*_*ter*_, and *KwIDP2*_*ter*_ are 150 bp downstream regions of *S*. *cerevisiae IDP3*, *IDP2*, *A*. *gossypii IDP2*, and *K*. *waltii IDP2* respectively: *e*.*g*. *ScIDP2*_*Pro*_ */ScIDP3/ScIDP3*_*Ter*_ represents the assembled fragment of the upstream region of *ScIDP2*, *ScIDP3* coding region, and the downstream region of *ScIDP3*. To introduce mutations (shown with underbars in primers sequences below) in the PTS1, or in the polyT region of *A*.*gossypii IDP2*, site-directed mutagenesis was performed by using pRS41K-*ScIDP3*_*Pro*_ */AgIDP2/AgIDP2*_*Ter*_ plasmid as a template with primer sets ptsdel_f (5’-GAAAAAGCAAGCATAATTATAGCCTAGGCTGCCT-3’) and ptsdel_r (5’-AGGCAGCCTAGGCTATAATTATGCTTGCTTTTTC-3’) for cPTS1 mutation, delt_f (5’-GGCTACAAGCGTCTTTTTGTGAATAAGAAAAAGC-3’) and delt_r (5’-GCTTTTTCTTATTCACAAAAAGACGCTTGTAGCC-3’) for single T deletion on the polyT, and tsyn_f (5’-GGCTACAAGCGTCTCTTCTGTGAATAAGAAAAAG-3’) and tsyn_r (5’-CTTTTTCTTATTCACAGAAGAGACGCTTGTAGCC-3’) for silent mutations on the polyT, generating pRS41K-*ScIDP3*_*Pro*_ */AgIDP2* ^*ΔAKL*^ */AgIDP2*_*Ter*_, pRS41K-*ScIDP3*_*Pro*_ */AgIDP2* ^*Δt*^ */AgIDP2*_*Ter*_, and pRS41K-*ScIDP3*_*Pro*_ */AgIDP2* ^*+silent*^ */AgIDP2*_*Ter*_, respectively. Replacement of the C-terminus of *S*.*cer IDP2* with the C-termini and 3’-UTRs of various genes was performed using Leu403 as the 5’ crossover point (as it comprises the last conserved residue in yeast *IDP*s), and the stop codon as the 3’ crossover.

### The growth assay for peroxisomal targeting

The peroxisome targeting potential of cryptic PST1 sequences was tested by measuring the growth rates of *ΔIdp3* strain complemented with various *IDP* genes in the petroselinate containing medium. The *IDP* constructs tested for targeting (native and chimeras alike) were cloned into a chromosomal pRS41K plasmid and transformed to the *ΔIdp3* strain. Cells were first grown on YPD media (1% yeast extract, 2% Bacto-peptone, 2% Glucose) for at least 18 hours, and then used to inoculate into the YP-petroselinate medium (1% yeast extract, 2% Bacto-peptone, 0.2% Tween-40, 0.1% petroselinate) at an initial OD_600_ 0.1. Growth was monitored by absorbance at 600 nm (error bars represent standard deviations of three independent cultures).

### Laboratory evolution experiments

The 100 bp region centered around the stop codon of *K*. *waltii IDP2* was randomly mutated by error-prone PCR using GeneMorph II random mutagenesis kit (Agilent technologies, CA) and integrated by MEGAWHOP cloning [57] into the *IDP2* encoding plasmid pRS41K-*ScIDP3*_*Pro*_ */KwIDP2/KwIDP2*_*Ter*_ (construction details above). Sequencing indicated an average mutation rate of ~1 mutation per gene. The plasmid library was transformed to the *ΔIdp3* strain. Cells were cultured for 300 hours in the YP-petroselinate medium as described above. The resulting culture with increased growth rates was plated on YPD plates and randomly chosen colonies were analyzed by DNA sequencing. Silent mutations were introduced in the plasmid pRS41K-*ScIDP3*_*Pro*_ */KwIDP2/KwIDP2*_*Ter*_ by site-direct mutagenesis. These plasmids were used to transform the *ΔIdp3* strain and test growths on petroselinate. Note that the *ΔIdp3* strain used here was derived from another adaptation experiment where the *ΔIdp3* strain complemented with *K*. *waltii IDP2* gene (pRS41K-*ScIDP3*_*Pro*_ */KwIDP2/KwIDP2*_*Ter*_) was cultured in the YP-petroselinate medium until spontaneously adapting.

### Construction of sequence alignment and phylogenic tree

*IDP* sequences were obtained from the Fungal Orthogroups Repository [58]. Sequence alignment was created by MUSCLE [59]. Maximum likelihood phylogenic trees were created by PhyML [60] based on the yeast species tree [58] by using the JTT substitution matrix.

### mRNA analysis

The *ΔIdp3* strain transformed by plasmid pRS41K-*ScIDP3*_*Pro*_ */AgIDP2/AgIDP2*_*Ter*_ was cultured in the YP media containing petroselinate until the mid-log phase (OD_600_ ~0.6). One mL culture was centrifuged and the collected cell pellet was subjected to total RNA extraction using total RNA extraction kit (Epicentre). The cDNA of *A*. *gossypii IDP2* was amplified from the total RNA by RT-PCR using gene-specific primers: xhoI_agidp500f (5’-ATTGGGTACCCTCGAGAGGACGGGGACAAGTCCAAG-3’) and agter_notIr (5’-CACCGCGGTGGCGGCCGCAGATATGCTAGACTAGTAATAAATAGACGC-3’). The amplified PCR products were subcloned into plasmid pRS41K using *XhoI/NotI*. The plasmids were transformed into *E*. *coli*. and 36 randomly selected colonies were subjected to DNA sequencing.

### Protein expression and purification

For expression in *S*.*cerevisiae* and purification, *A*.*gossypii IDP2*’s ORF plus 150 bps of the 3’-UTR region was amplified by PCR (from plasmid pRS41K-*ScIDP3*_*Pro*_ */AgIDP2/AgIDP2*_*Ter*_) with primers encoding an N-terminal Histidine-tag. The DNA fragment was subcloned into plasmid pFA6a-nat [61] using *XhoI/SpeI* sites, for expression and the strong constitutive *TEF2* promoter and *ADH1*’s terminator. The resulting construct including the promoter and terminator was excised using *SacI* sites and subcloned into plasmid pRS42H [56], a multicopy 2μ-based yeast plasmid, generating pRS42H-*His*:*AgIDP2/AgIDP2*_*Ter*_. This plasmid was transformed into the adaptive wild-type strain (*WT*). For purification of histidine-tagged protein, transformants were pre-cultivated in YPD for 18 hours at 30°C, transferred to YPD at a starting OD 0.05. The culture was harvested at OD 2.2 after 22 hours incubation at 30°C. Harvested cells were resuspended in the two-fold cell volumes of lysis buffer (50 mM potassium phosphate (pH 8.0), 300 mM sodium chloride, 2 mM sodium citrate, 10 mM Imidazole, 10% glycerol, 1 mM DTT, 0.1% Triton-100, 1% protease inhibitor cocktail (Sigma)) and lysed by voltex with the same cell volume of glass beads (425–600 nm, Sigma; G8772) and subsequent sonication. Cell debris was removed by centrifugation for 30 min at 11,500 rpm, and the supernatant was passed through a open column containing Ni-NTA resin. The column was washed with 30-fold resin volumes of wash buffer (50 mM potassium phosphate (pH 8.0), 300 mM sodium chloride, 2 mM sodium citrate, 20 mM Imidazole, 10% glycerol). The proteins were finally eluted with elution buffer (50 mM potassium phosphate (pH 8.0), 300 mM sodium chloride, 2 mM sodium citrate, 250 mM Imidazole, 10% glycerol). The eluted samples were buffer-exchanged with PBS (137 mM sodium chloride, 2.7 mM potassium chloride, 1.76 mM potassium dihydrogenphosphate, 10 mM disodium hydrogenphosphate, pH 7.4) containing 10% glycerol and 2 mM sodium citrate and concentrated by ultrafiltration (Vivaspin 500-10K, GE). Typical yield was ~ 0.2 mg/L at >90% purity as judged by SDP-PAGE.

### Mass spectrometry

Microcapillary reverse phase liquid chromatography (LC) was performed with a nanoAcquity UPLC system (nUPLC) (Waters Corp.), using the HEMA/EDMA (Hexyl methacrylate/Ethylene glycol dimethacrylate 60/40 v/v) monolithic column prepared in-house, as previously described [62]. Proteins (5μl of 50 ng/mL) were loaded onto the column and separated using a linear gradient of 20% to 60% solvent B over 40 min, at a flow rate of 10–15 μl/min, at 60°C. Solvent A was water + 0.05% formic acid+ 0.035% Trifluoroacetic acid and solvent B was acetonitrile + 0.05% formic acid+ 0.035% Trifluoroacetic acid. The LC eluant was sprayed into a Qstar XL mass spectrometer (MDS Sciex, Canada) by means of an electrospray ion (ESI) source. The following experimental parameters were used: capillary 5.3 kV, declustering potential of 40 V, focusing potential of 200 V, and second declustering potential of 20 V. The covered mass range was 500–5,000 m/z. Minimal smoothing and centering parameters were used. Spectra were calibrated using a solution of Reserpine (1 μM). The experimental masses of both the original and alternative isoforms corresponded to the theoretical values minus 130 Da due to the removal of the initial methionine [63].

### Colocalization experiment by fluorescent microscopy

Peroxisomal localization was confirmed by mating strains expressing Pex14p fused to GFP and the C-terminal proteins of interest fused to mCherry. The reference haploid strain (By4742: *MATα*, *his3Δ1*, *leu2Δ0*, *met15Δ0*, *ura3Δ0*) expressing Pex14p C-terminally tagged with GFP was constructed as described before [64]. The C-terminal fragments from *A*.*gos IDP2* or *S*.*cer PYC1* were inserted to the C-terminus of mCherry in plasmid pbs69_PRX_tdh3_mCherry, derived from pBS35 (yeast resource center), by whole plasmid PCR. The plasmid was digested in the *Mfe1* site and integrated into the *TDH3* promoter site of the adapted wild-type strain (*WT*) by homologous recombination. The transformed haploid *WT* strain was selected in the presence of Hygromycin (300 μg/ml), and analysed for positive RFP signal by fluorescent microscopy. The RFP-tagged strains were then mated with the GFP-tagged reference strain in SD medium lacking Histidine containing Hygromycin, and the resulting diploid strains were visualized by fluorescent microscopy (Nikon Ecripse Ti, Japan). Images were taken using a cooled CCD camera with an exposure time of 40–300 ms and processed using ImageJ (National Institutes of Health).

### Computational search for PTS1 in 3’-UTR regions

The motif sets used for PTS1 search were built from 20 known peroxisomal genes (13 genes containing the canonical PTS1, 7 genes either containing resembled PTS1 or C-terminus responsible for its localization) from Saccaromyces Genome Database. Each position in the motif was defined as the union of amino acids that appeared in these genes ((S/A/C/E/I/H/Q)-(K/R/H)-(L/F)-stop), in order to minimize the possibility for false negative. Searched were the motifs starting within the first 30 bps of the 3’ UTR, excluding the one disturbed by an additional stop codon. This search was performed in four genomes (downloaded from the Saccaromyces Genome Database): *S*. *cerevisiae* (S288C reference genome version R64), *S*. *pardoxus* (strain NRRL Y-17217), *S*. *bayanus* (WashU version) and *S*. *mikatae* (strain IFO1815). For each cryptic PTS1 candidate, 12 amino acids upstream to the PTS1 (including it) was extracted and manually scored by using the PTS1 predictor (http://mendel.imp.ac.at/pts1/ PTS1predictor.jsp) [45]. The cryptic PTS1 appeared in the coding frame were scored by replacing the original stop codons UAA/UAG and UGA with glutamine and arginine, respectively, according to the known stop codon read-through [65,66].

## Supporting Information

S1 Fig The most significant event in the divergence of *IDP3* is the acquisition of the PTS1. (A) The *ΔIdp3* strain was complemented with plasmids carrying the following genes: *IDP3*, *IDP2*, *IDP2* ^*+CKL*^; *null* relates to the *ΔIdp3* strain with no plasmid. *IDP2* ^*+CKL*^ was constructed by modifying the *IDP2* gene by replacing its stop codon with *IDP3*’s PTS1—a CKL tripeptide followed by a stop codon (CKL_*_). These genes were cloned into a chromosomal plasmid and transformed into the *ΔIdp3* strain. Transformed cells were grown in YP medium with petroselinate as the main carbon source. While wild-type *IDP2* showed no complementation of *ΔIdp3* growth, *IDP2* ^*+CKL*^ fully complemented it, as does *IDP3*. (B) *IDP3* gene driven by *IDP2* or *IDP3* promoter (300 bp upstream region) complemented *ΔIdp3* growth on petroselinate. The growths are not significantly different, indicating no significant divergence in the *IDP2* and *IDP3* promoters. Note that both promoter regions contain oleate response element, binding site for the oleate-specific transcriptional activator Pip2 [67]. Error bars are standard deviations of three independent cultures.
(TIF)

S2 Fig The pre-duplication *IDP2* genes have functional cryptic PTS1 motifs. (A) Various cryptic PTS1 of the pre-duplication *IDP2* genes were fused with *S*. *cerevisiae IDP2* and tested by plasmid complementation of the *ΔIdp3* growth in YP medium with petroselinate. The C-terminal 9 amino acids of *S*.*cer IDP2* was replaced with the C-termini of the different pre-duplication *IDP2* genes, including cryptic PTS1 at their 3’UTRs. All the cryptic PTS1s of pre-duplication *IDP2* genes but *K*. *waltii* are functional. Note that *K*. *lactice* does not have a cryptic PTS1 in the 3’UTR thus no growth was seen. Error bars are standard deviations of three independent cultures. (B) The full length of *K*. *waltii IDP2* gene, spanning from the start ATG codon up to 150 bps downstream the stop codon, was used for the plasmid complementation of the *ΔIdp3* growth in the YP-petroselinate medium. Shown is the C-terminal sequence around the stop codon (black rectangle) including the cryptic PTS1 (green rectangle). Fast growth on petroselinate was induced by a single T deletion (highlighted in grey) just before the stop codon, thus introducing the cryptic motif (AKL*) within the coding frame (*K*.*wal IDP2* ^*Δt*^; black growth curve).
(TIF)

S3 Fig Cryptic PTS1 of *S*. *cerevisiae PYC1* is functional for peroxisomal targeting when revealed by a frameshit. (A) A PTS1 signature (-SHL*) is conserved at 3’UTR of *PYC1* genes among all four yeast species. The motives were found at +1-shifted coding frame in *S*. *cerevisiae*, *S*. *pardoxus*, and *S*. *bayanus*, and on frame in *S*. *mikatae*. These motives were scored and evaluated by extracting 12 amino acids upstream to the motives with the PTS1 predictor. Note that the original stop codon of *S*. *mikatae PYC1* was replaced to serine residue (TGA → TCA) for the scoring. (B) The mCherry was C-terminally tagged with the C-terminal *S*.*cer PYC1* fragment (the last 11 amino acids and the 3’UTR ending with SHL*) and co-expressed with Pex14-GFP fusion. The cellular localization was observed by fluorescent microscopy after the cells were grown in SD plates for two days. The frame-shifted sequence (shown in Fig 3B) by the single nucleotide deletion showed clear peroxisomal localization while the native sequence showing cytosolic localization.
(TIF)

S4 Fig Cryptic PTS1-like motif (-NKF*) of *S*. *cerevisiae ACO2* is functional and evolvable for peroxisomal targeting when revealed by a frameshift. (A) The motif is found at +1-shifted coding frame in *S*. *cerevisiae* and evaluated as ‘Not targeted’ with the PTS1 predictor; nevertheless we further tested the functionality. (B) The C-terminal fragment of *S*.*cer ACO2* (last 10 amino acids *plus* the 3’UTR ending with NKF*) was fused to the C-terminus of *S*.*cer IDP2* and used for complementation of *ΔIdp3* growth on petroselinate. As can be seen, the motif confers weak yet reproducible growth beyond the *IDP2* control strain when revealed in-frame by a single base deletion upstream the stop codon (*S*.*cer ACO2* ^*ΔA*^; the deleted A is shown in Fig 3C). Error bars are standard deviations of three independent cultures. (C) Upon serial transfers to a fresh petrosalinate medium (5-fold dilutions), a marked increase in growth rate was observed (Fig 3C). Single colonies were randomly isolated from the 2^nd^ transferred culture (at 900 h) and sequenced. All 7 sequenced clones possessed a single nonsynonymous mutation in the PTS1-like motif converting it to NKL.
(TIF)

S1 Table The number of genes containing potential PTS1 motifs in *Saccharomyces* species. (PDF)

S2 Table List of yeast strains used in this study. (PDF)

S3 Table Genes containing potential PTS1 motifs in *Saccharomyces* species. (PDF)

## References

[1] Khersonsky O, Tawfik DS (2010) Enzyme promiscuity: a mechanistic and evolutionary perspective. Annu Rev Biochem 79: 471–505. 10.1146/annurev-biochem-030409-143718 20235827

[2] O'Brien PJ, Herschlag D (1999) Catalytic promiscuity and the evolution of new enzymatic activities. Chem Biol 6: R91–R105. 10099128 10.1016/S1074-5521(99)80033-7

[3] Jensen RA (1976) Enzyme recruitment in evolution of new function. Annu Rev Microbiol 30: 409–425. 791073 10.1146/annurev.mi.30.100176.002205

[4] Masel J, Bergman A (2003) The evolution of the evolvability properties of the yeast prion [PSI+]. Evolution 57: 1498–1512. 12940355 10.1111/j.0014-3820.2003.tb00358.x

[5] Tawfik DS (2010) Messy biology and the origins of evolutionary innovations. Nat Chem Biol 6: 692–696. 10.1038/nchembio.441 20852602

[6] Whitehead DJ, Wilke CO, Vernazobres D, Bornberg-Bauer E (2008) The look-ahead effect of phenotypic mutations. Biol Direct 3: 18 10.1186/1745-6150-3-18 18479505 PMC2423361

[7] Willensdorfer M, Burger R, Nowak MA (2007) Phenotypic mutation rates and the abundance of abnormal proteins in yeast. PLoS Comput Biol 3: e203 18039025 10.1371/journal.pcbi.0030203 PMC2082502

[8] Parker J (1989) Errors and alternatives in reading the universal genetic code. Microbiol Rev 53: 273–298. 2677635 10.1128/mr.53.3.273-298.1989 PMC372737

[9] Ellis N, Gallant J (1982) An estimate of the global error frequency in translation. Mol Gen Genet 188: 169–172. 6759868 10.1007/BF00332670

[10] Ohno S (1970) Evolution by gene duplication: Springer.

[11] Piatigorsky J (2007) Gene Sharing and Evolution: The Diversity of Protein Functions. Cambridge, Massachusetts, USA; London, UK: Harvard Univ. Press.

[12] Force A, Lynch M, Pickett FB, Amores A, Yan YL, et al (1999) Preservation of duplicate genes by complementary, degenerative mutations. Genetics 151: 1531–1545. 10101175 10.1093/genetics/151.4.1531 PMC1460548

[13] Hughes AL (1994) The evolution of functionally novel proteins after gene duplication. Proc Biol Sci 256: 119–124. 8029240 10.1098/rspb.1994.0058

[14] Soskine M, Tawfik DS (2010) Mutational effects and the evolution of new protein functions. Nat Rev Genet 11: 572–582. 10.1038/nrg2808 20634811

[15] Wroe R, Chan HS, Bornberg-Bauer E (2007) A structural model of latent evolutionary potentials underlying neutral networks in proteins. Hfsp J 1: 79–87. 10.2976/1.2739116/10.2976/1 19404462 PMC2645552

[16] Amitai G, Gupta RD, Tawfik DS (2007) Latent evolutionary potentials under the neutral mutational drift of an enzyme. Hfsp J 1: 67–78. 10.2976/1.2739115/10.2976/1 19404461 PMC2645560

[17] Hittinger CT, Carroll SB (2007) Gene duplication and the adaptive evolution of a classic genetic switch. Nature 449: 677–681. 17928853 10.1038/nature06151

[18] Sayou C, Monniaux M, Nanao MH, Moyroud E, Brockington SF, et al (2014) A promiscuous intermediate underlies the evolution of LEAFY DNA binding specificity. Science 343: 645–648. 10.1126/science.1248229 24436181

[19] Coyle SM, Flores J, Lim WA (2013) Exploitation of latent allostery enables the evolution of new modes of MAP kinase regulation. Cell 154: 875–887. 10.1016/j.cell.2013.07.019 23953117 PMC3787944

[20] Bridgham JT, Carroll SM, Thornton JW (2006) Evolution of hormone-receptor complexity by molecular exploitation. Science 312: 97–101. 37000978 10.1681/01.asn.0000926836.46869.e5

[21] Des Marais DL, Rausher MD (2008) Escape from adaptive conflict after duplication in an anthocyanin pathway gene. Nature 454: 762–765. 10.1038/nature07092 18594508

[22] Marques AC, Vinckenbosch N, Brawand D, Kaessmann H (2008) Functional diversification of duplicate genes through subcellular adaptation of encoded proteins. Genome Biol 9: R54 10.1186/gb-2008-9-3-r54 18336717 PMC2397506

[23] Conant GC, Wolfe KH (2008) Turning a hobby into a job: how duplicated genes find new functions. Nat Rev Genet 9: 938–950. 10.1038/nrg2482 19015656

[24] Innan H, Kondrashov F (2010) The evolution of gene duplications: classifying and distinguishing between models. Nat Rev Genet 11: 97–108. 10.1038/nrg2689 20051986

[25] Bergthorsson U, Andersson DI, Roth JR (2007) Ohno's dilemma: evolution of new genes under continuous selection. Proc Natl Acad Sci U S A 104: 17004–17009. 17942681 10.1073/pnas.0707158104 PMC2040452

[26] Kisslov I, Naamati A, Shakarchy N, Pines O (2014) Dual-targeted proteins tend to be more evolutionarily conserved. Mol Biol Evol 31: 2770–2779. 10.1093/molbev/msu221 25063438

[27] Regev-Rudzki N, Pines O (2007) Eclipsed distribution: a phenomenon of dual targeting of protein and its significance. Bioessays 29: 772–782. 17621655 10.1002/bies.20609

[28] Ast J, Stiebler AC, Freitag J, Bolker M (2013) Dual targeting of peroxisomal proteins. Front Physiol 4: 297 10.3389/fphys.2013.00297 24151469 PMC3798809

[29] Williams CC, Jan CH, Weissman JS (2014) Targeting and plasticity of mitochondrial proteins revealed by proximity-specific ribosome profiling. Science 346: 748–751. 10.1126/science.1257522 25378625 PMC4263316

[30] Freitag J, Ast J, Bolker M (2012) Cryptic peroxisomal targeting via alternative splicing and stop codon read-through in fungi. Nature 485: 522–525. 10.1038/nature11051 22622582

[31] Schueren F, Lingner T, George R, Hofhuis J, Dickel C, et al (2015) Peroxisomal lactate dehydrogenase is generated by translational readthrough in mammals. Elife 3: e03640. 10.7554/eLife.03640 PMC4359377 25247702

[32] Byrne KP, Wolfe KH (2005) The Yeast Gene Order Browser: combining curated homology and syntenic context reveals gene fate in polyploid species. Genome Res 15: 1456–1461. 16169922 10.1101/gr.3672305 PMC1240090

[33] Ihmels J, Levy R, Barkai N (2004) Principles of transcriptional control in the metabolic network of Saccharomyces cerevisiae. Nat Biotechnol 22: 86–92. 14647306 10.1038/nbt918

[34] Henke B, Girzalsky W, Berteaux-Lecellier V, Erdmann R (1998) IDP3 encodes a peroxisomal NADP-dependent isocitrate dehydrogenase required for the beta-oxidation of unsaturated fatty acids. J Biol Chem 273: 3702–3711. 9452501 10.1074/jbc.273.6.3702

[35] Gould SJ, Keller GA, Hosken N, Wilkinson J, Subramani S (1989) A conserved tripeptide sorts proteins to peroxisomes. J Cell Biol 108: 1657–1664. 2654139 10.1083/jcb.108.5.1657 PMC2115556

[36] Brocard C, Hartig A (2006) Peroxisome targeting signal 1: is it really a simple tripeptide? Biochim Biophys Acta 1763: 1565–1573. 17007944 10.1016/j.bbamcr.2006.08.022

[37] Lu Q, McAlister-Henn L (2010) Peroxisomal localization and function of NADP+-specific isocitrate dehydrogenases in yeast. Arch Biochem Biophys 493: 125–134. 10.1016/j.abb.2009.10.011 19854152 PMC2812674

[38] Stiebler AC, Freitag J, Schink KO, Stehlik T, Tillmann BA, et al (2014) Ribosomal readthrough at a short UGA stop codon context triggers dual localization of metabolic enzymes in Fungi and animals. PLoS Genet 10: e1004685 10.1371/journal.pgen.1004685 25340584 PMC4207609

[39] Rockah-Shmuel L, Toth-Petroczy A, Sela A, Wurtzel O, Sorek R, et al (2013) Correlated occurrence and bypass of frame-shifting insertion-deletions (InDels) to give functional proteins. PLoS Genet 9: e1003882 10.1371/journal.pgen.1003882 24204297 PMC3812077

[40] Tamas I, Wernegreen JJ, Nystedt B, Kauppinen SN, Darby AC, et al (2008) Endosymbiont gene functions impaired and rescued by polymerase infidelity at poly(A) tracts. Proc Natl Acad Sci U S A 105: 14934–14939. 10.1073/pnas.0806554105 18815381 PMC2567471

[41] Wagner LA, Weiss RB, Driscoll R, Dunn DS, Gesteland RF (1990) Transcriptional slippage occurs during elongation at runs of adenine or thymine in Escherichia coli. Nucleic Acids Res 18: 3529–3535. 2194164 10.1093/nar/18.12.3529 PMC331007

[42] Warnecke T, Hurst LD (2011) Error prevention and mitigation as forces in the evolution of genes and genomes. Nat Rev Genet 12: 875–881. 10.1038/nrg3092 22094950

[43] Drummond DA, Wilke CO (2009) The evolutionary consequences of erroneous protein synthesis. Nat Rev Genet 10: 715–724. 10.1038/nrg2662 19763154 PMC2764353

[44] Kunze M, Hartig A (2013) Permeability of the peroxisomal membrane: lessons from the glyoxylate cycle. Front Physiol 4: 204 10.3389/fphys.2013.00204 23966945 PMC3743077

[45] Neuberger G, Maurer-Stroh S, Eisenhaber B, Hartig A, Eisenhaber F (2003) Prediction of peroxisomal targeting signal 1 containing proteins from amino acid sequence. J Mol Biol 328: 581–592. 12706718 10.1016/s0022-2836(03)00319-x

[46] Aharoni A, Gaidukov L, Khersonsky O, Mc QGS, Roodveldt C, et al (2005) The 'evolvability' of promiscuous protein functions. Nat Genet 37: 73–76. 15568024 10.1038/ng1482

[47] Kondrashov FA (2005) In search of the limits of evolution. Nat Genet 37: 9–10. 15624013 10.1038/ng0105-9

[48] Small I, Wintz H, Akashi K, Mireau H (1998) Two birds with one stone: genes that encode products targeted to two or more compartments. Plant Mol Biol 38: 265–277. 9738971

[49] Danpure CJ (1995) How can the products of a single gene be localized to more than one intracellular compartment? Trends Cell Biol 5: 230–238. 14732127 10.1016/s0962-8924(00)89016-9

[50] Kochetov AV (2008) Alternative translation start sites and hidden coding potential of eukaryotic mRNAs. Bioessays 30: 683–691. 10.1002/bies.20771 18536038

[51] Chang KJ, Wang CC (2004) Translation initiation from a naturally occurring non-AUG codon in Saccharomyces cerevisiae. J Biol Chem 279: 13778–13785. 14734560 10.1074/jbc.M311269200

[52] Rajon E, Masel J (2011) Evolution of molecular error rates and the consequences for evolvability. Proc Natl Acad Sci U S A 108: 1082–1087. 10.1073/pnas.1012918108 21199946 PMC3024668

[53] True HL, Lindquist SL (2000) A yeast prion provides a mechanism for genetic variation and phenotypic diversity. Nature 407: 477–483. 11028992 10.1038/35035005

[54] Brachmann CB, Davies A, Cost GJ, Caputo E, Li J, et al (1998) Designer deletion strains derived from Saccharomyces cerevisiae S288C: a useful set of strains and plasmids for PCR-mediated gene disruption and other applications. Yeast 14: 115–132. 9483801 10.1002/(SICI)1097-0061(19980130)14:2<115::AID-YEA204>3.0.CO;2-2

[55] Wach A, Brachat A, Pohlmann R, Philippsen P (1994) New heterologous modules for classical or PCR-based gene disruptions in Saccharomyces cerevisiae. Yeast 10: 1793–1808. 7747518 10.1002/yea.320101310

[56] Taxis C, Knop M (2006) System of centromeric, episomal, and integrative vectors based on drug resistance markers for Saccharomyces cerevisiae. Biotechniques 40: 73–78. 16454043 10.2144/000112040

[57] Miyazaki K (2003) Creating random mutagenesis libraries by megaprimer PCR of whole plasmid (MEGAWHOP). Methods Mol Biol 231: 23–28. 12824598 10.1385/1-59259-395-X:23

[58] Wapinski I, Pfeffer A, Friedman N, Regev A (2007) Natural history and evolutionary principles of gene duplication in fungi. Nature 449: 54–61. 17805289 10.1038/nature06107

[59] Edgar RC (2004) MUSCLE: multiple sequence alignment with high accuracy and high throughput. Nucleic Acids Res 32: 1792–1797. 15034147 10.1093/nar/gkh340 PMC390337

[60] Guindon S, Gascuel O (2003) A simple, fast, and accurate algorithm to estimate large phylogenies by maximum likelihood. Syst Biol 52: 696–704. 14530136 10.1080/10635150390235520

[61] Wach A, Brachat A, Alberti-Segui C, Rebischung C, Philippsen P (1997) Heterologous HIS3 marker and GFP reporter modules for PCR-targeting in Saccharomyces cerevisiae. Yeast 13: 1065–1075. 9290211 10.1002/(SICI)1097-0061(19970915)13:11<1065::AID-YEA159>3.0.CO;2-K

[62] Rozen S, Tieri A, Ridner G, Stark AK, Schmaler T, et al Exposing the subunit diversity within protein complexes: a mass spectrometry approach. Methods 59: 270–277. 10.1016/j.ymeth.2012.12.013 23296018

[63] Kimura Y, Saeki Y, Yokosawa H, Polevoda B, Sherman F, et al (2003) N-Terminal modifications of the 19S regulatory particle subunits of the yeast proteasome. Arch Biochem Biophys 409: 341–348. 12504901 10.1016/s0003-9861(02)00639-2

[64] Huh WK, Falvo JV, Gerke LC, Carroll AS, Howson RW, et al (2003) Global analysis of protein localization in budding yeast. Nature 425: 686–691. 14562095 10.1038/nature02026

[65] Edelman I, Culbertson MR (1991) Exceptional codon recognition by the glutamine tRNAs in Saccharomyces cerevisiae. Embo J 10: 1481–1491. 2026145 10.1002/j.1460-2075.1991.tb07668.x PMC452811

[66] Chittum HS, Lane WS, Carlson BA, Roller PP, Lung FD, et al (1998) Rabbit beta-globin is extended beyond its UGA stop codon by multiple suppressions and translational reading gaps. Biochemistry 37: 10866–10870. 9692979 10.1021/bi981042r

[67] Rottensteiner H, Kal AJ, Filipits M, Binder M, Hamilton B, et al (1996) Pip2p: a transcriptional regulator of peroxisome proliferation in the yeast Saccharomyces cerevisiae. Embo J 15: 2924–2934. 8670793 PMC450233

