# Supplementary figures and images for: The Evolutionary Potential of Phenotypic Mutations
Source: PLoS Genet. 2015 Aug 5;11(8):e1005445. doi: 10.1371/journal.pgen.1005445 (PMC4526572; doi:10.1371/journal.pgen.1005445)

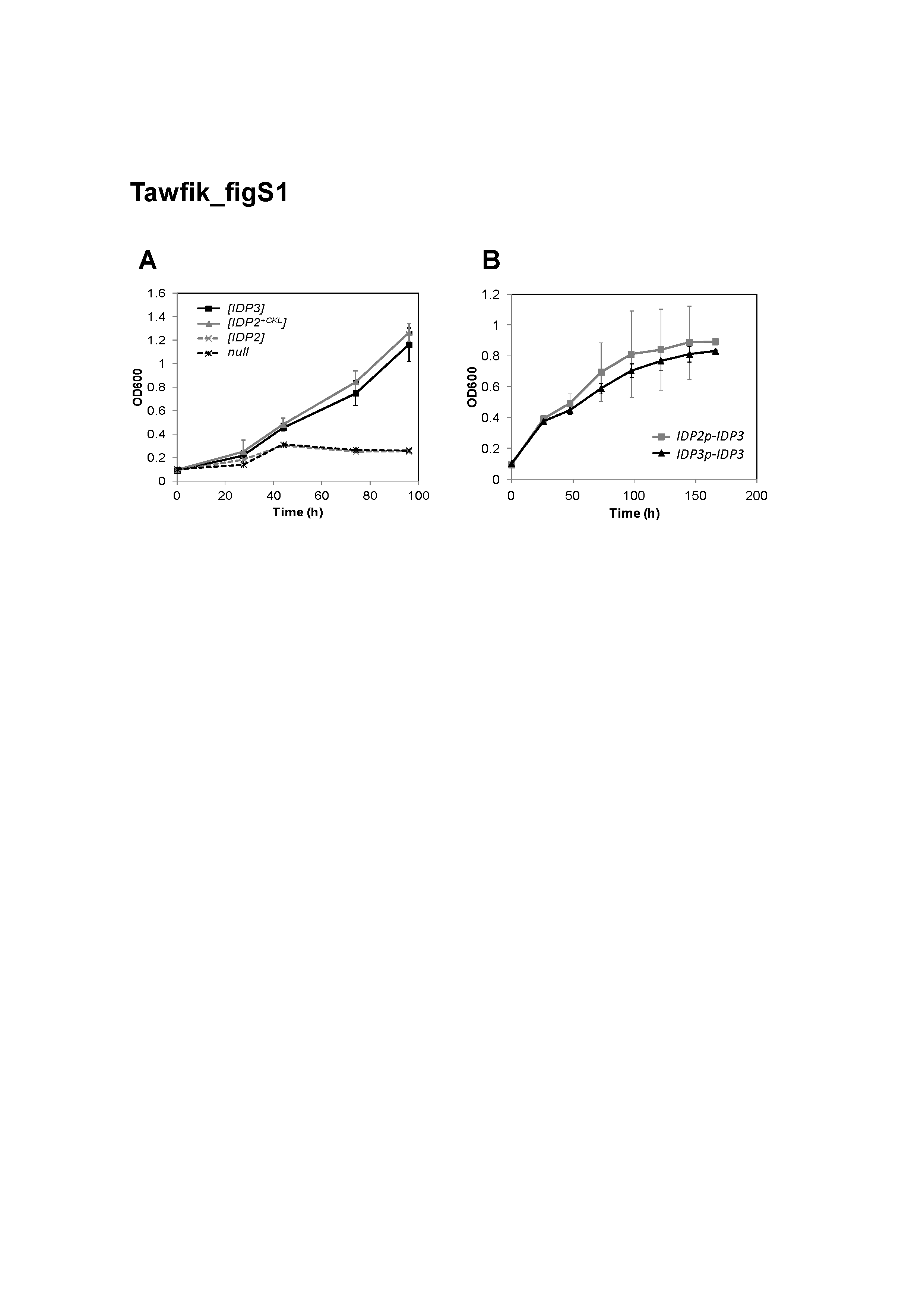

Supplement: S1 Fig — (A) The ΔIdp3 strain was complemented with plasmids carrying the following genes: IDP3, IDP2, IDP2 +CKL; null relates to the ΔIdp3 strain with no plasmid. IDP2 +CKL was constructed by modifying the IDP2 gene by replacing its stop codon with IDP3’s PTS1—a CKL tripeptide followed by a stop codon (CKL*). These genes were cloned into a chromosomal plasmid and transformed into the ΔIdp3 strain. Transformed cells were grown in YP medium with petroselinate as the main carbon source. While wild-type IDP2 showed no complementation of ΔIdp3 growth, IDP2 +CKL fully complemented it, as does IDP3. (B) IDP3 gene driven by IDP2 or IDP3 promoter (300 bp upstream region) complemented ΔIdp3 growth on petroselinate. The growths are not significantly different, indicating no significant divergence in the IDP2 and IDP3 promoters. Note that both promoter regions contain oleate response element, binding site for the oleate-specific transcriptional activator Pip2 [67]. Error bars are standard deviations of three independent cultures. (TIF) [file pgen.1005445.s001.tif]

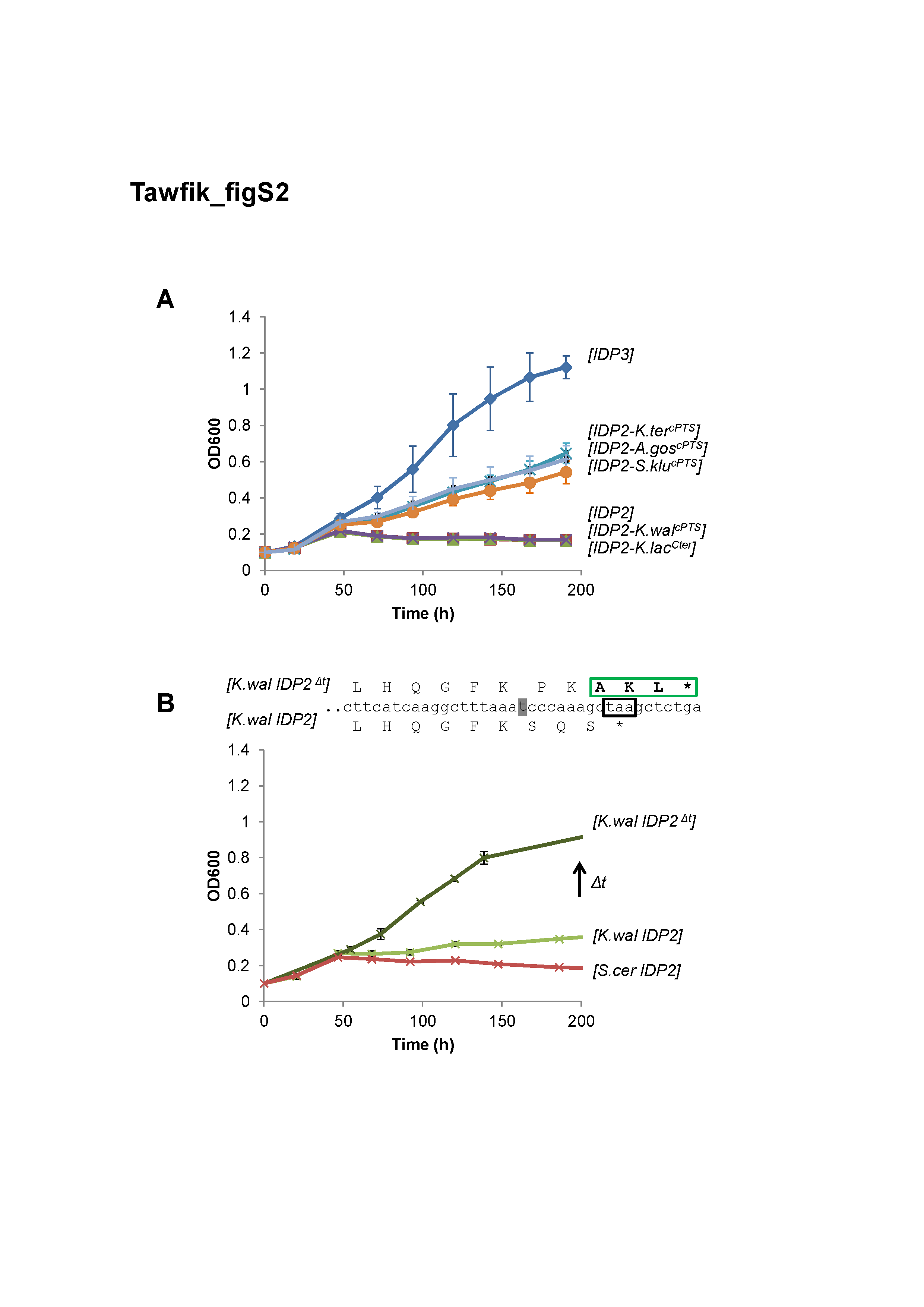

Supplement: S2 Fig — (A) Various cryptic PTS1 of the pre-duplication IDP2 genes were fused with S. cerevisiae IDP2 and tested by plasmid complementation of the ΔIdp3 growth in YP medium with petroselinate. The C-terminal 9 amino acids of S.cer IDP2 was replaced with the C-termini of the different pre-duplication IDP2 genes, including cryptic PTS1 at their 3’UTRs. All the cryptic PTS1s of pre-duplication IDP2 genes but K. waltii are functional. Note that K. lactice does not have a cryptic PTS1 in the 3’UTR thus no growth was seen. Error bars are standard deviations of three independent cultures. (B) The full length of K. waltii IDP2 gene, spanning from the start ATG codon up to 150 bps downstream the stop codon, was used for the plasmid complementation of the ΔIdp3 growth in the YP-petroselinate medium. Shown is the C-terminal sequence around the stop codon (black rectangle) including the cryptic PTS1 (green rectangle). Fast growth on petroselinate was induced by a single T deletion (highlighted in grey) just before the stop codon, thus introducing the cryptic motif (AKL*) within the coding frame (K.wal IDP2 Δt; black growth curve). (TIF) [file pgen.1005445.s002.tif]

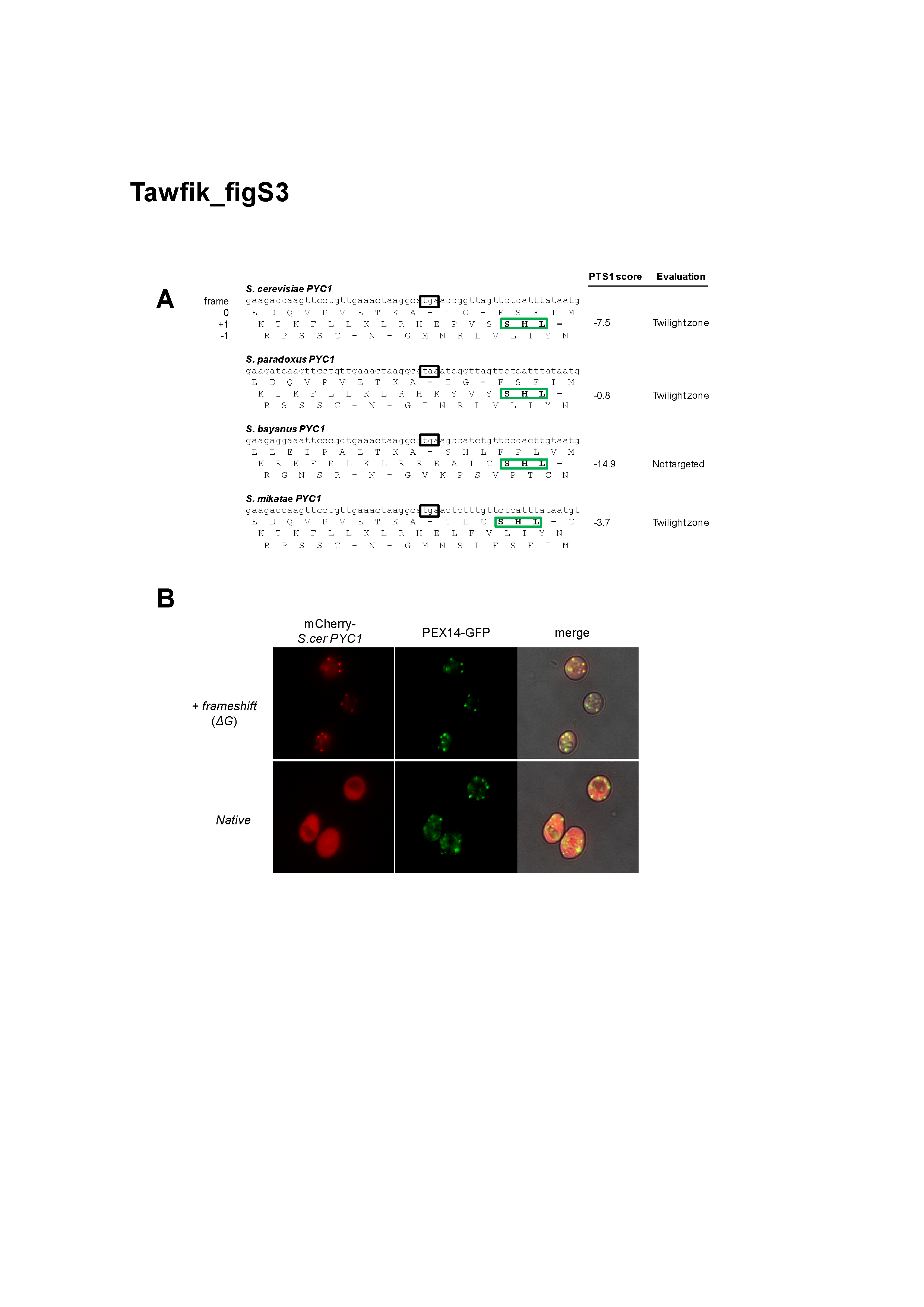

Supplement: S3 Fig — (A) A PTS1 signature (-SHL*) is conserved at 3’UTR of PYC1 genes among all four yeast species. The motives were found at +1-shifted coding frame in S. cerevisiae, S. pardoxus, and S. bayanus, and on frame in S. mikatae. These motives were scored and evaluated by extracting 12 amino acids upstream to the motives with the PTS1 predictor. Note that the original stop codon of S. mikatae PYC1 was replaced to serine residue (TGA → TCA) for the scoring. (B) The mCherry was C-terminally tagged with the C-terminal S.cer PYC1 fragment (the last 11 amino acids and the 3’UTR ending with SHL*) and co-expressed with Pex14-GFP fusion. The cellular localization was observed by fluorescent microscopy after the cells were grown in SD plates for two days. The frame-shifted sequence (shown in Fig 3B) by the single nucleotide deletion showed clear peroxisomal localization while the native sequence showing cytosolic localization. (TIF) [file pgen.1005445.s003.tif]

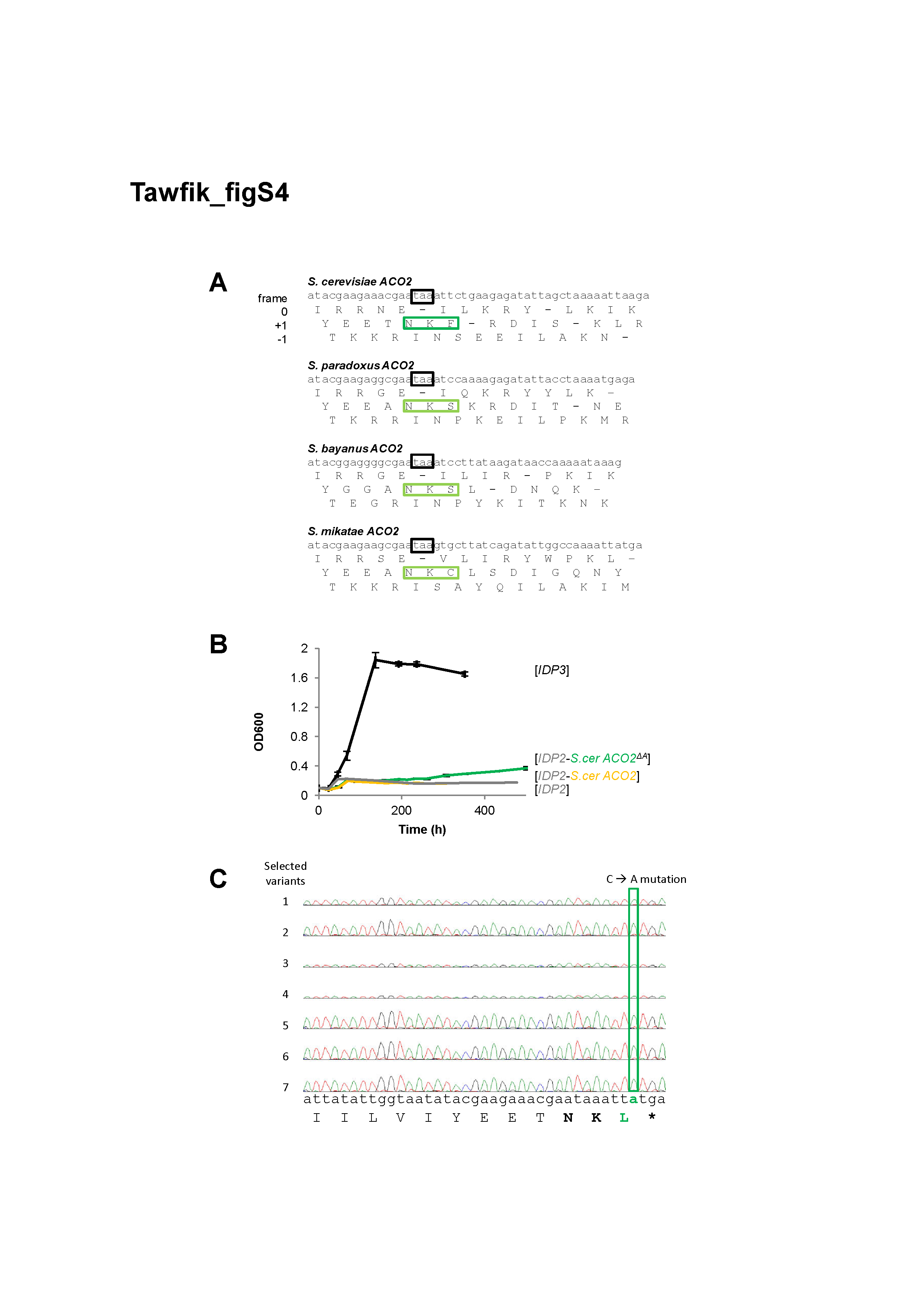

Supplement: S4 Fig — (A) The motif is found at +1-shifted coding frame in S. cerevisiae and evaluated as ‘Not targeted’ with the PTS1 predictor; nevertheless we further tested the functionality. (B) The C-terminal fragment of S.cer ACO2 (last 10 amino acids plus the 3’UTR ending with NKF*) was fused to the C-terminus of S.cer IDP2 and used for complementation of ΔIdp3 growth on petroselinate. As can be seen, the motif confers weak yet reproducible growth beyond the IDP2 control strain when revealed in-frame by a single base deletion upstream the stop codon (S.cer ACO2 ΔA; the deleted A is shown in Fig 3C). Error bars are standard deviations of three independent cultures. (C) Upon serial transfers to a fresh petrosalinate medium (5-fold dilutions), a marked increase in growth rate was observed (Fig 3C). Single colonies were randomly isolated from the 2nd transferred culture (at 900 h) and sequenced. All 7 sequenced clones possessed a single nonsynonymous mutation in the PTS1-like motif converting it to NKL. (TIF) [file pgen.1005445.s004.tif]
