# Supplementary material for: The Evolutionary Potential of Phenotypic Mutations
Source: PLoS Genet. 2015 Aug 5;11(8):e1005445. doi: 10.1371/journal.pgen.1005445 (PMC4526572; doi:10.1371/journal.pgen.1005445)
Supplement: S1 Table — (PDF) [file pgen.1005445.s005.pdf]

Table S1. The number of genes containing potential PTS1 motifs in *Saccharomyces* species

	S. cerevisiae	*S. paradoxus*	*S. bayanus*	*S. mikatae*
Total # of genes	6650	4788	4492	4525
*Genes with cryptic PTS1	70	46	36	48
†Cryptic PTS1 motifs disturbed by stop codon	24	21	14	17
‡Predicted as "targeted"	6	3	4	2
Predicted as "twilight zone"	7	5	4	7

*PTS1 motif was defined as (S/A/C/E/I/H/Q)-(K/R/H)-(L/F)-stop according to 20 peroxisomal proteins with PTS1 from Saccharomyces genome database: http://www.yeastgenome.org/cgi-bin/GO/goTerm.pl?goid= GO:5777. The motif starting within 30 bp downstream from the stop codon was searched.

†Cryptic PTS1 containing stop codons between the end of genes and the PTS1 were counted. The genes with cryptic PTS1 not disturbed by stop codons are listed in **Table S3**. Some genes possess conserved intact motifs among species as follows; YGL062W (*PYC1*) in all species; YLL027W (*ISA1*) in three species; YBR014C (*GRX7*), YGL086W (*MAD1*), YDR238C (*SEC26*), YNL052W (*COX5A*), YNL156C (*NSG2*), and YPL152W (*RRD2*) in two species.

‡The 12 amino acids upstream from the cryptic PTS1 (including it) was extracted and manually scored using the PTS1 predictor: http://mendel.imp.ac.at/pts1/PTS1predictor.jsp. The genes predicted as either targeted or twilight zone are listed in **Table S3**.
