# Supplementary material for: The Evolutionary Potential of Phenotypic Mutations
Source: PLoS Genet. 2015 Aug 5;11(8):e1005445. doi: 10.1371/journal.pgen.1005445 (PMC4526572; doi:10.1371/journal.pgen.1005445)
Supplement: S2 Table — (PDF) [file pgen.1005445.s006.pdf]

Table S2. List of yeast strains used in this study

Strain	Genotype	Resistance	Reference
BY4741	*MATa, his3Δ1, leu2Δ0, met15Δ0, ura3Δ0*	-	[54]
WT	*MATa, his3Δ1, leu2Δ0, met15Δ0, ura3Δ0, idp3:: IDP3-kanMX4*	Kan^R	This study
ΔIdp3	*MATa, his3Δ1, leu2Δ0, met15Δ0, ura3Δ0, idp3:: hphNT1*	Hyg^R	This study
IDP3	*ΔIdp3* transformed by pRS41K-$ScIDP3_{Pro}$/ScIDP3/$ScIDP3_{Ter}$	Kan^R, Hyg^R	This study
$IDP2_{Pro}$-IDP3	*ΔIdp3* transformed by pRS41K-$ScIDP2_{Pro}$/ScIDP3/$ScIDP3_{Ter}$	Kan^R, Hyg^R	This study
$IDP2^{+CKL}$	*ΔIdp3* transformed by pRS41K-$ScIDP3_{Pro}$/$ScIDP2^{+CKL}$/$ScIDP3_{Ter}$	Kan^R, Hyg^R	This study
IDP2	*ΔIdp3* transformed by pRS41K-$ScIDP3_{Pro}$/ScIDP2/$ScIDP2_{Ter}$	Kan^R, Hyg^R	This study
A.gos IDP2	*ΔIdp3* transformed by pRS41K-$ScIDP3_{Pro}$/AgIDP2/$AgIDP2_{Ter}$	Kan^R, Hyg^R	This study
A.gos $IDP2^{ΔAKL}$	*ΔIdp3* transformed by pRS41K-$ScIDP3_{Pro}$/$AgIDP2^{ΔAKL}$/$AgIDP2_{Ter}$	Kan^R, Hyg^R	This study
A.gos $IDP2^{Δt}$	*ΔIdp3* transformed by pRS41K-$ScIDP3_{Pro}$/$AgIDP2^{Δt}$/$AgIDP2_{Ter}$	Kan^R, Hyg^R	This study
A.gos $IDP2^{+silent}$	*ΔIdp3* transformed by pRS41K-$ScIDP3_{Pro}$/$AgIDP2^{+silent}$/$AgIDP2_{Ter}$	Kan^R, Hyg^R	This study
His:AgIDP2	*WT* transformed by pRS42H-His/AgIDP2/$AgIDP2_{Ter}$	Kan^R, Hyg^R	This study
His:$AgIDP2^{Δt}$	*WT* transformed by pRS42H-His/$AgIDP2^{Δt}$/$AgIDP2_{Ter}$	Kan^R, Hyg^R	This study
His:$AgIDP2^{+silent}$	*WT* transformed by pRS42H-His/$AgIDP2^{+silent}$/$AgIDP2_{Ter}$	Kan^R, Hyg^R	This study
