# Supplementary material for: The Evolutionary Potential of Phenotypic Mutations
Source: PLoS Genet. 2015 Aug 5;11(8):e1005445. doi: 10.1371/journal.pgen.1005445 (PMC4526572; doi:10.1371/journal.pgen.1005445)
Supplement: S3 Table — (PDF) [file pgen.1005445.s007.pdf]

Table S3. Genes containing potential PTS1 motifs in *Saccharomyces* species

S. cerevisiae

gene name	ORF name	distance from stop	PTS1 seq	frame	stop codon	last 12 aa seq	PTS1 predictor	prediction
YAL066W	YAL066W	13	QKF*	0	TAG	TSCI*KITVQKF* --> TSCIQKITVQKF*	-13.079	not targeted
URA7	YBL039C	2	SKF*	1	TAA	PAKTNSTFKSKF*	11.222	targeted
GRX7	YBR014C	1	IRL*	0	TAG	ANSQSESA*IRL* --> ANSQSESAQIRL*	-11.172	not targeted
NPL4	YBR170C	1	ERL*	0	TAG	VMILQESG*ERL* --> VMILQESGQERL*	-23.728	not targeted
PGS1	YCL004W	20	CRL*	1	TAG	NFRFYSIIVCRL*	-31.754	not targeted
TAH1	YCR060W	14	EHF*	1	TGA	TTGPEKREREHF*	-10.106	not targeted
YDL121C	YDL121C	30	QKL*	2	TAA	MEVIFAIVLQKL*	-75.066	not targeted
RPS29B	YDL061C	6	ERL*	2	TAA	WFPQVQISQERL*	-21.904	not targeted
SEC26	YDR238C	21	CKL*	2	TGA	CLNHPELMQCKL*'	-13.889	not targeted
SSN2	YDR443C	24	AHL*	2	TAA	INFFRQRFPAHL*	-28.487	not targeted
PUG1	YER185W	10	IKF*	0	TAG	QTLSN*IIYIKF* --> QTLSNQIIYIKF*	-21.986	not targeted
DOC1	YGL240W	6	CRL*	2	TAA	PGCYITLIICRL*	-38.6	not targeted
SUA5	YGL169W	1	IKL*	0	TAA	AANNCIQF*IKL*--> AANNCIQFQIKL*	-22.599	not targeted
MAD1	YGL086W	7	IHF*	0	TAA	EQRQAK*VYIHF* --> EQRQAKQVYIHF*	-24.468	not targeted
PYC1	YGL062W	11	SHL*	1	TGA	MKLRHEPVSSHL*'	-7.46	Twilight zone
RPL26B	YGR034W	5	ARL*	1	TAA	ERVVNWNKLARL*	-7.368	Twilight zone
PHB1	YGR132C	6	EKL*	2	TAA	AFEHWPLSLEKL*	-21.371	not targeted
YGR291C	YGR291C	5	CKL*	1	TAA	GPRKFPDKSCKL*	-0.425	Twilight zone
YHR020W	YHR020W	8	SHL*	1	TAG	CSVVLISCISHL*	-36.554	not targeted
COX6	YHR051W	23	QRL*	1	TAA	LNNKEKATTQRL*	-5.667	Twilight zone
PAN5	YHR063C	27	IHL*	2	TGA	KMRGYQPEHIHL*	-20.869	not targeted
YIL068W-A	YIL068W-A	24	HRL*	2	TGA	MIKNFKIPLHRL*	-17.871	not targeted
YIL012W	YIL012W	12	QKL*	2	TAA	KISFVSEVTQKL*	-4.573	Twilight zone
YIR007W	YIR007W	14	QKL*	1	TAA	ALSAKQKLNQKL*	0.139	targeted
NUC1	YJL208C	8	EKL*	1	TGA	LQKKGIECTEKL*	-14.629	not targeted

AIM22	YJL046W	18	IHL*	2	TAA	LHITLMNISIHL*	-41.687	not targeted
YJR151W-A	YJR151W-A	24	IHL*	2	TGA	MTKIPFGLQIHL*	-33.953	not targeted
MPH3	YJR160C	11	HHL*	1	TAG	PLQRNSNVSHHL*	-2.719	Twilight zone
MRP17	YKL003C	9	HRF*	2	TAG	RLSIHLGIAHRF*	-18.102	not targeted
OSH6	YKR003W	26	CKL*	1	TAG	SVYVGYDICCKL*	-16.402	not targeted
ISA1	YLL027W	11	HHF*	1	TAA	RVSWFKNLLHHF*	-37.27	not targeted
DNM1	YLL001W	13	SHF*	0	TAA	SNIL*LHNSSHF* --> SNILQLHNSSHF*	-16.134	not targeted
ECM30	YLR436C	2	HKF*	1	TAG	IPLEGCIDRHKF*	-12.448	not targeted
GAB1	YLR459W	6	SRL*	2	TAA	IEGNTNLTTSRL*	1.578	targeted
RRN11	YML043C	8	SKL*	1	TGA	MKTQVSEQTSKL*	5.183	targeted
CGI121	YML036W	18	IKL*	2	TAG	GCVDKRYLTIKL*	-27.227	not targeted
YNL208W	YNL208W	19	CHF*	0	TGA	RW*MAQQSECHF* --> RWRMAQQSECHF*	-19.327	not targeted
NSG2	YNL156C	3	SKF*	2	TAA	RKISIFSLKSKF*	-17.775	not targeted
COX5A	YNL052W	7	QKF*	0	TGA	SQVQSK*TGQKF* --> SQVQSKRTGQKF*	-1.034	Twilight zone
YNL018C	YNL018C	6	QKF*	2	TGA	LNFCTSVNRQKF*	-16.417	not targeted
DBP6	YNR038W	26	IHL*	1	TAA	NFSLSTFASIHL*	-27.202	not targeted
HAL9	YOL089C	16	QRL*	0	TAA	DYE*TVPCLQRL* --> DYEQTVPCLQRL*	-12.311	not targeted
TIR2	YOR010C	1	SHF*	0	TAA	MAAAAMLL*SHF* --> MAAAAMLLQSHF*	-60.543	not targeted
RPO31	YOR116C	4	SRF*	0	TAA	EAALKAN*SSRF* --> EAALKANQSSRF*	3.608	targeted
YPL277C	YPL277C	1	CHL*	0	TGA	CQRCRRNC*CHL* --> CQRCRRNCRCHL*	0.807	targeted
ATP6	Q0085	9	IKL*	2	TAA	CSILTLNYKIKL*	-34.173	not targeted

S. paradoxus	ORF name	distance from stop	PTS1 seq	frame	stop codon	last 12 aa seq	PTS1 predictor	prediction
	YAR002W'	3	'IKF*'	2	TGA	FQVFIHFLIIKF*	-100.882	not targeted
	YBL063W'	8	'SRF*'	1	TAA	IKNFISNEISRF*	-1.126	Twilight zone

YBR014C'	1	'IRL*'	0	TAG	ANSQSESA*IRL*' --> ANSQSESAQIRL*	-11.172	not targeted
YBR139W'	21	'SRF*'	2	TGA	ELIVSALSMSRF*	-5.319	Twilight zone
YBR260C'	24	'SHL*'	2	TAA	ISILLIILLSHL*	-102.189	not targeted
YDR011W'	19	'HHF*'	0	TAA	KQ*CGLQENHHF* --> KQQCGLQENHHF*	-16.237	not targeted
YDR051C'	4	'QRF*'	0	TAA	CEGDLTT*NQRF* --> CEGDLTTQNQRF*	-10.511	not targeted
YDR238C'	21	'CKL*'	2	TGA	CLNDSESRKCKL*	10.046	targeted
YDR333C'	1	'ERL*'	0	TAA	ESNENEAQ*ERL*' --> ESNENEAQQERL*	-10.641	not targeted
YDR384C'	26	'IKF*'	1	TAA	KEYETKAKLIKF*	-16.034	not targeted
YGL086W'	6	'IHF*'	2	TAA	GTTTGQIMYIHF*	-32.769	not targeted
YGL071W'	13	'IKF*'	0	TAG	KRED*HINEIKF*' --> KREDQHINEIKF*	-17.314	not targeted
YGL062W'	11	'SHL*'	1	TAA	MKLRHKSVSSHL*	-0.81	Twilight zone
YIL043C'	4	'EKL*'	0	TAA	EDQVFVF*DEKL*' --> EDQVFVFQDEKL*	-43.819	not targeted
YIL011W'	2	'CHF*'	1	TGA	MVQPLCSYDCHF*	-32.58	not targeted
YJL020C'	21	'EKL*'	2	TAA	MVILLKTFEEKL*	-69.763	not targeted
YKL122C'	4	'IHL*'	0	TAA	NKGKKKR*AIHL*' --> NKGKKKRQAIHL*	-13.334	not targeted
YKL092C'	22	'SHF*'	0	TGA	T*PKMFNSHSHF*' --> TRPKMFNSHSHF*	-9.748	Twilight zone
YLL027W'	10	'HHF*'	0	TAA	ESFMV*NLLHHF*' --> ESFMVQNLLHHF*	-29.36	not targeted
YLR111W'	13	'AHL*'	0	TGA	GSRV*WFRHAHL*' --> GSRVRWFRHAHL*	-36.983	not targeted
YLR421C'	2	'SRF*'	1	TAA	MWMLICKIKSRF*	-46.303	not targeted
YNL052W'	7	'QKF*'	0	TGA	SQVQSK*TDQKF*' --> SQVQSKRTDQKF*	-2.633	Twilight zone
YNL023C'	28	'AHF*'	0	TGA	NIIVYHYLYAHF*	-64.64	not targeted
YOL117W'	22	'CRF*'	0	TAG	I*NIEQTRSCRF*' --> IQNIEQTRSCRF*	1.965	targeted

YPL152W'	7	'ARL*'	0	TAG	KPIPFD*KKARL*' --> KPIPFDQKKARL*	8.397	targeted

S. mikatae

ORF name	distance from stop	PTS1 seq	frame	stop codon	last 12 aa seq	PTS1 predictor	prediction
YBR009C'	4	'SHF*'	0	TAA	TLYGFGG*SSHF*' --> TLYGFGGQSSHF*	-4.174	Twilight zone
YBR223C'	7	'QKL*'	0	TAG	ARQEDN*YRQKL*' --> ARQEDNQYRQKL*	-1.636	Twilight zone
YCR020W-B'	21	'CRF*'	2	TAA	KLRKIVSRNCRF*	-8.694	Twilight zone
YDR072C'	16	'IKL*'	0	TAG	PLA*TIGYQIKL*' --> PLAQTIGYQIKL*	-21.466	not targeted
YDR141C'	20	'CKL*'	1	TAA	YHNPVLEFFCKL*	-24.751	not targeted
YDR180W'	6	'SHL*'	2	TAA	KTRKRYVNMSHL*	-19.577	not targeted
YDR449C'	11	'IHL*'	1	TAA	SLANFKCVIIHL*	-33.084	not targeted
YDR518W'	25	'AHL*'	0	TAA	*SINKMVYKAHL*' --> QSINKMVYKAHL*	-22.793	not targeted
YEL002C'	13	'HHL*'	0	TAG	KKTN*CTFSHHL*' --> KKTNQCTFSHHL*	-17.591	not targeted
YER139C'	4	'AKF*'	0	TGA	GDFTKED*TAKF*' --> GDFTKEDRTAKF*	5.267	targeted
YGL147C'	8	'IRL*'	1	TAA	SLLKIYKLFIRL*	-57.064	not targeted
YGL062W'	10	'SHL*'	0	TGA	VETKA*TLCSHL*' --> VETKARTLCSHL*	-3.4	twilight zone
YGL033W'	28	'EHF*'	0	TGA	TFIQDEIGVEHF*	-26.872	not targeted
YGR158C'	24	'ERL*'	2	TGA	MIILAVKTNERL*	-64.539	not targeted
YGR279C'	5	'ARL*'	1	TAG	GVFYPMNRIARL*	-7.111	Twilight zone
YHL030W'	6	'EKL*'	2	TGA	IKKHWYMTIEKL*	-59.906	not targeted
YKL207W'	16	'ARL*'	0	TAA	QYM*VVATYARL*' --> QYMQVVATYARL*	-12.343	not targeted
YLL029W'	20	'ERL*'	1	TAA	HYKFEAQYAERL*	-30.128	not targeted
YLL027W'	11	'HRF*'	1	TAA	RVLWFKNHLHRF*'	-48.147	not targeted
YML100W'	13	'SKF*'	0	TAA	IIED*FLLISKF*' --> IIEDQFLLISKF*	-38.3	not targeted

YMR255W'	16	'ARF*'	0	TAA	MRI*SFSLYARF*' --> MRIQSFSLYARF*	-17.42	not targeted
YNL211C'	5	'IRF*'	1	TGA	DWGTALNDRIRF*	-15.691	not targeted
YNL200C'	10	'SHL*'	0	TGA	QILKL*WKPSHL*' --> QILKLRWKPSHL*	-29.842	not targeted
YNL156C'	3	'SKF*'	2	TAA	'RKISIFSLSSKF*'	-20.277	not targeted
YNL073W'	4	'CKF*'	0	TAA	VDDVNRQ*NCKF*' --> VDDVNRQQNCKF*	-2.94	twilight zone
YNL005C'	25	'IHL*'	0	TGA	*FFFSYARYIHL*' --> RFFFSYARYIHL*	-36.301	not targeted
YOR016C'	10	'ERL*'	0	TAG	QKNYV*TAFERL*'--> QKNYVQTAFERL*	-18.99	not targeted
YOR113W'	13	'QHF*'	0	TAG	NYKS*PLLQQHF*' --> NYKSQPLLQQHF*	-20.683	not targeted
YPL231W'	25	'IHL*'	0	TAG	*NELYIILSIHL*' --> QNELYIILSIHL*	-61.707	not targeted
YPL174C'	5	'ARL*'	1	TAA	HIKKYGNNLARL*	2.758	targeted
YPL152W'	7	'AHL*'	0	TAA	KPIPFD*KHAHL*' --> KPIPFDQKHAHL*	-1.098	twilight zone

S. bayanus

ORF name	distance from stop	PTS1 seq	frame	stop codon	last 12 aa seq	PTS1 predictor	prediction
YBL098W'	14	'CRL*'	1	TAA	ESVPKSAGSCRL*	7.271	targeted
YBR053C'	4	'IRL*'	0	TGA	TKPQPLY*SIRL*' --> TKPQPLYRSIRL*	-20.406	not targeted
YER012W'	28	'EKL*'	0	TGA	KIFRENTRKEKL*	-5.989	Twilight zone
YER101C'	29	'CRF*'	1	TGA	KLCLFLSKFCRF*	-27.946	not targeted
YFL045C'	8	'CHL*'	1	TAA	MKYSTYNNICHL*	-12.379	not targeted
YGL062W'	11	'SHL*'	1	TGA	'MKLRREAICSHL*'	-14.896	not targeted
YHR069C'	21	'HKL*'	2	TGA	QLKKKIINLHKL*	-15.319	not targeted
YHR112C'	8	'IRL*'	1	TGA	SCKMLLEYCIRL*	-40.116	not targeted
YJR061W'	25	'QKF*'	0	TAA	*GTLSSFSKQKF*' --> QGTLSSFSKQKF*	-0.113	twilight zone

YLL052C'	19	'QHL*'	0	TGA	KD*INTKFLQHL*' --> KDRINTKFLQHL*	-17.823	not targeted
YML018C'	17	'CRL*'	1	TGA	QTTDSKKKPCRL*	2.71	targeted
YMR106C'	24	'QRF*'	2	TAG	LALEKLENTQRF*	-16.059	not targeted
YMR178W'	6	'QKL*'	2	TAA	ILQSGEVKSQKL*	-2.364	Twilight zone
YOL059W'	11	'SHF*'	1	TAG	TSRTNRRLQSHF*	-0.632	Twilight zone
YOR062C'	25	'IRF*'	0	TGA	*FFCHIIVYIRF*' -> RFFCHIIVYIRF*	-75.272	not targeted
YOR270C'	5	'QRL*'	1	TAA	MLPALQAKGQRL*	-20.277	not targeted
YOR285W'	11	'SKL*'	1	TAG	TNLVFSYIHSKL*	-25.895	not targeted
YOR352W'	8	'IRL*'	1	TAA	TVPTRSNLSIRL*	-12.962	not targeted
YPL164C'	9	'IHF*'	2	TAA	SRNKRLRVLIHF*	-26.814	not targeted
YPL104W'	8	'SRL*'	1	TGA	MAIRKSEDKSRL*	5.891	targeted
YPR001W'	23	'IHL*'	1	TAG	YSTTSYKFRIHL*	-24.719	not targeted
YPR185W'	17	'CKL*'	1	TGA	KKVEKGNILCKL*	4.006	targeted
